# Supplementary material for: Adherens Junction Length during Tissue Contraction Is Controlled by the Mechanosensitive Activity of Actomyosin and Junctional Recycling
Source: Dev Cell. 2018 Nov 19;47(4):453–463.e3. doi: 10.1016/j.devcel.2018.10.025 (PMC6291457; doi:10.1016/j.devcel.2018.10.025)
Supplement: Document S1. Figures S1–S6 [file mmc1.pdf]

Developmental Cell, Volume *47*

Supplemental Information

Adherens Junction Length during Tissue Contraction Is Controlled by the Mechanosensitive Activity of Actomyosin and Junctional Recycling

Angughali Sumi, Peran Hayes, Arturo D'Angelo, Julien Colombelli, Guillaume Salbreux, Kai Dierkes, and Jérôme Solon

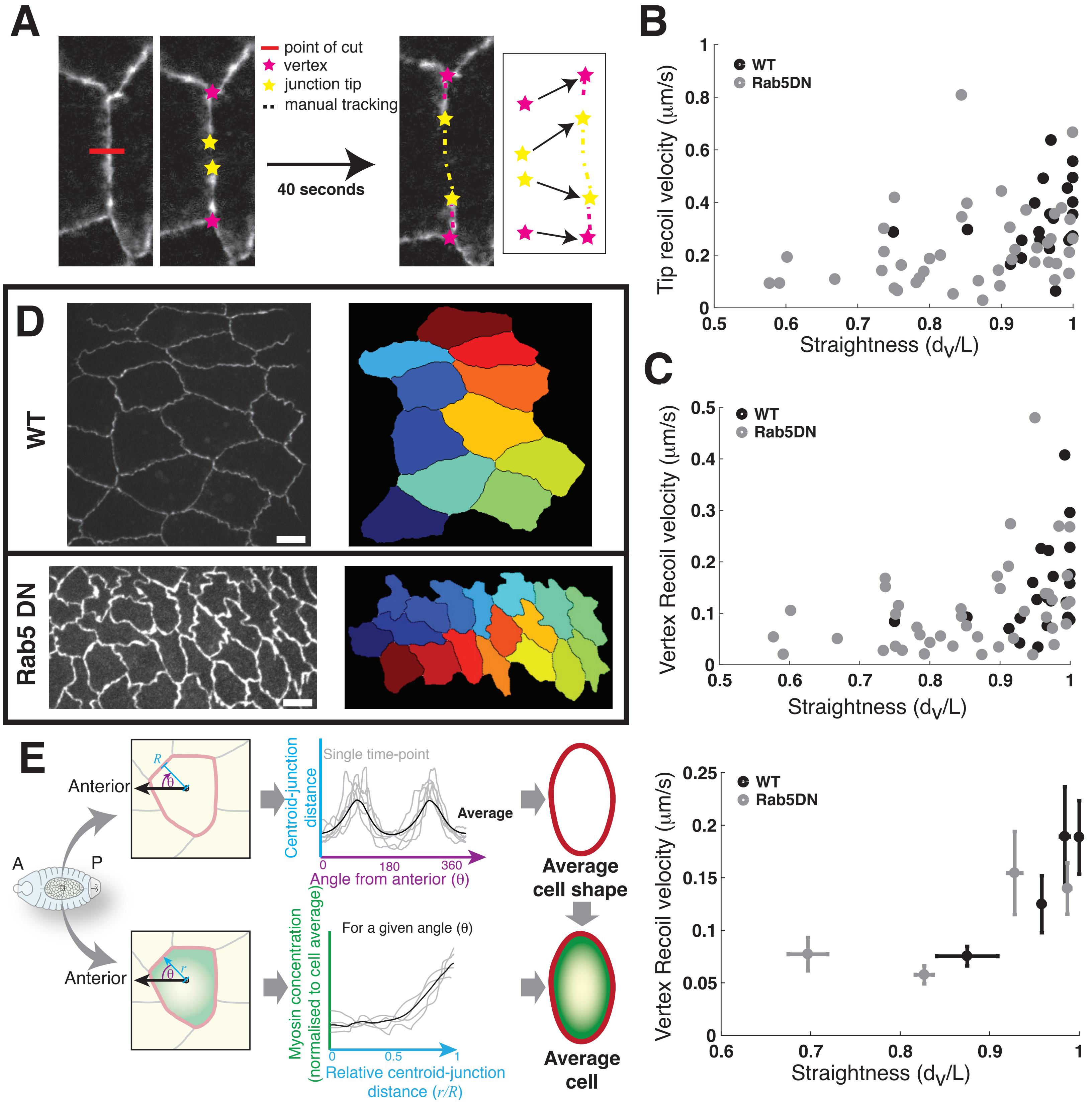
A
point of cut
vertex
junction tip
manual tracking
40 seconds
B
WT
Rab5DN
Tip recoil velocity (μm/s)
Straightness (dV/L)
C
WT
Rab5DN
Vertex Recoil velocity (μm/s)
Straightness (dV/L)
D
WT
Rab5 DN
E
Anterior
R
θ
r
A
P
Centroid-junction distance
Single time-point
Average
Angle from anterior (θ)
0
180
360
Average cell shape
Myosin concentration (normalised to cell average)
For a given angle (θ)
Relative centroid-junction distance (r/R)
0
0.5
1
Average cell
WT
Rab5DN
Vertex Recoil velocity (μm/s)
Straightness (dV/L)

Figure S1. Methodologies for laser dissection, cell segmentation and Average cell generation (related to figure 1 and Figure 2).

(A) Schematic explaining the methodology used to extract the initial recoil velocity from laser dissection experiments. Tips of junctions and vertices are tracked manually after laser dissection over a time period of 40 seconds, to extract the distance from their starting position as function of time. Onto this, an exponential fit is performed to extract the initial recoil velocity.

(B) Graph showing the initial recoil velocity after laser dissection of tips of junctions as a function of the straightness of the junction before laser dissection for WT and Rab5DN expressing cells. We observe an increase in recoil velocity when reaching a straightness close to 1. Each point is for an individual junction, and shows the average of the initial recoil velocities of the two cut ends.

(C) (Top) Graph showing the initial recoil velocity after laser dissection of the vertices of dissected junctions as a function of the straightness of the junction before laser dissection for WT and Rab5DN expressing cells. We observe an increase in recoil velocity when reaching a straightness close to 1. Each point shows the average of the initial recoil velocities of the two vertices of an individual junction. (Bottom) Graph showing the averaged initial recoil velocity of vertices after laser dissection corresponding to the graph above. Each point is an average of 5-6 junctions for WT and 10-11 junctions for Rab5DN. Error bars show standard errors of the mean. N_{WT}=22 junctions and N_{Rab5DN}=43 junctions.

(D) Images showing examples of WT and Rab5DN expressing AS cells tagged with Tomato-Ecadherin and their respective segmentation performed using Packing Analyzer V2.0 and a Python script (see methods).

(E) Schematic explaining the methodology to generate an average cell. The cells are oriented along the AP axis. With a radial scanning, the distance *R* between the cell contour and the center of mass is detected for each angle , to reconstruct an average contour (Top). Using the same radial scan, myosin levels along the line *r* from the center of mass cell to the cell contour are detected and an average for each angle is generated and scaled onto the average contour.

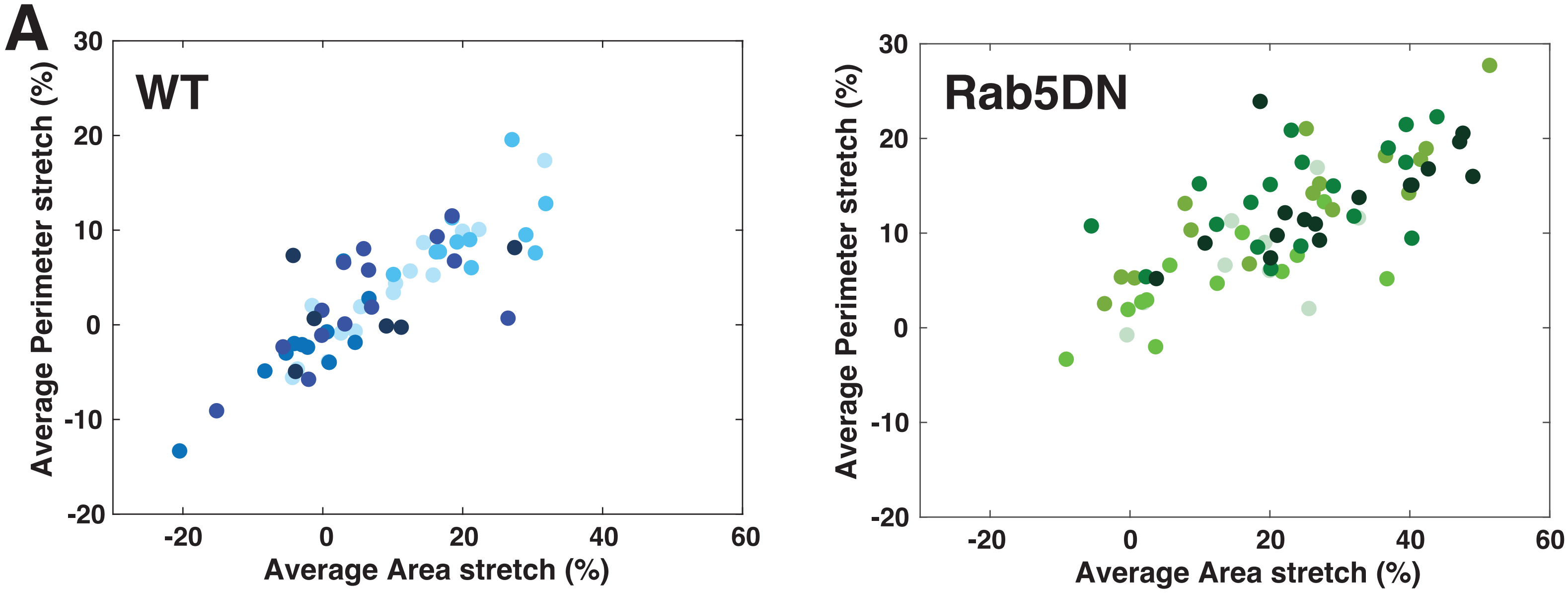

	Area stretch	Perimeter stretch
Embryo 1	9.5 ± 10 %	3.5 ± 6 %
Embryo 2	22 ± 7 %	9.5 ± 4 %
Embryo 3	-2.1 ± 7.1 %	-2.5 ± 4.7 %
Embryo 4	6 ± 11 %	2.4 ± 6 %
Embryo 5	6.5 ± 12.2 %	1.8 ± 5 %

	Area stretch	Perimeter stretch
Embryo 1	17.2 ± 11 %	7.2 ± 5.6 %
Embryo 2	12 ±13.5 %	4.6 ± 4.7 %
Embryo 3	23.3 ± 17.6 %	13.5 ± 6.8 %
Embryo 4	23.9 ± 13.7 %	13.8 ± 5.2 %
Embryo 5	29.7 ± 13.7 %	13.4 ± 5.1 %

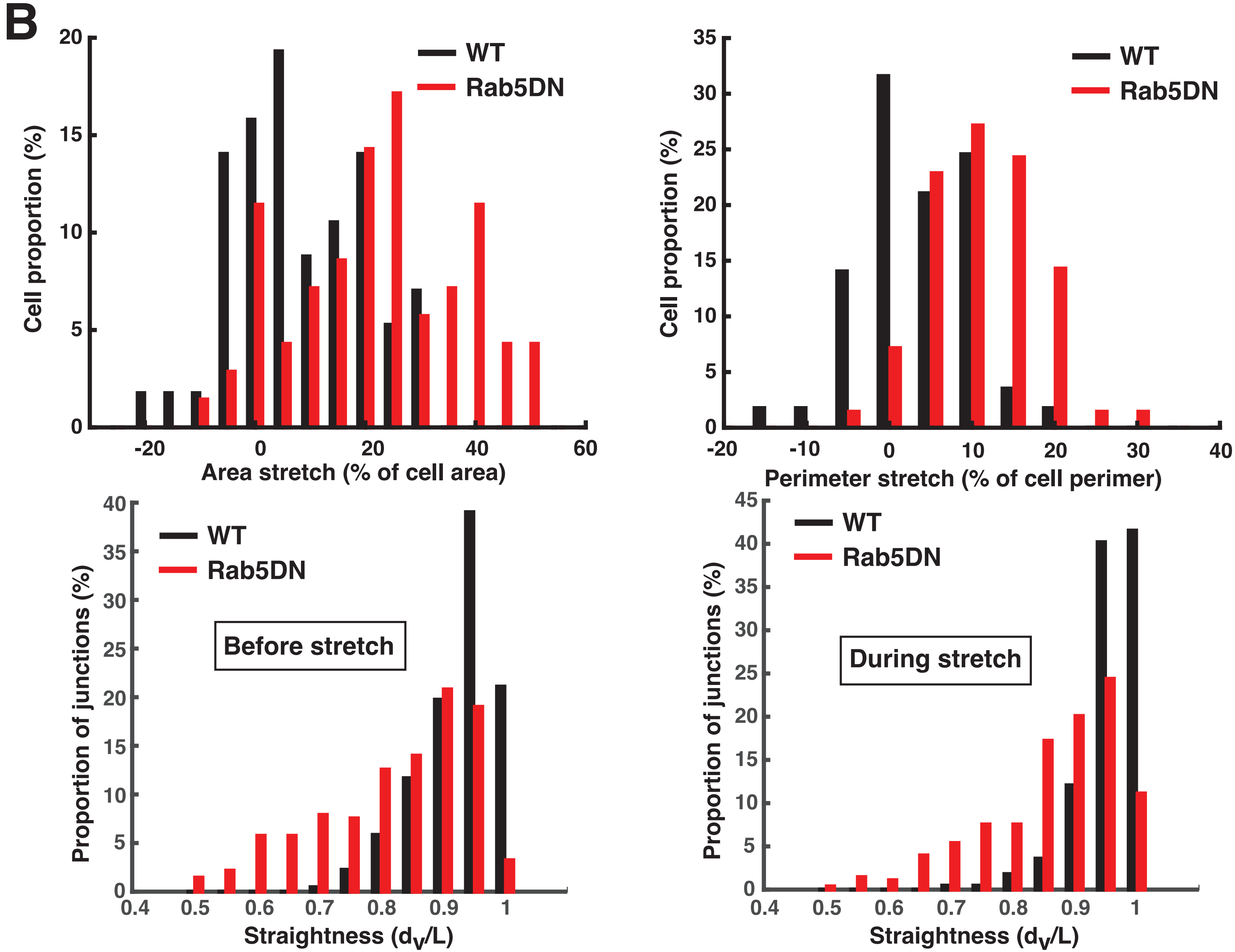

Figure S2. Amplitude of stretch performed by squeezing embryos (related to figure 2 and 3).

(A) (Top) Graphs showing the average perimeter stretch as a function of the average area stretch during our squeezing experiments for WT (Left) and Rab5DN expressing cells (Right). Average area/perimeter stretch was calculated as the average area/perimeter within the first minute after the stretch onset divided by the average area/perimeter over the last 10-15 minutes before stretch. Each dot represents one cell and each color, one embryo (corresponding to the embryos in the tables below). (Bottom) Tables showing the average stretch and standard deviation per embryo analyzed for WT (Left) and Rab5DN expressing cells (Right).

(B) (Top-Left) Distribution of the increase in average area per cell obtained during stretch experiments for WT and Rab5DN expressing cells. In WT, we generate an average area increase of $8.4 \pm 12\%$ and in Rab5, we generate an average area increase of $22 \pm 15\%$.

(Top-Right) Distribution of the increase in average perimeter per cell obtained during stretch experiments for WT and Rab5DN expressing cells. In WT, we generate an average perimeter increase of $3 \pm 6.4\%$ and in Rab5, we generate an average perimeter increase of $11 \pm 6.5\%$.

(Bottom-Left and Right) Graphs showing the distribution of junctional straightness for WT and Rab5DN expressing cells within the minute before (Left) and during (Right) stretch.

(Bottom-Left) Before stretch is applied; WT junctions exhibit an average straightness of 0.92 ± 0.06, compared to 0.82 ± 0.12 for junctions of Rab5DN expressing cells. This difference is due to the presence of increased ruffles in Rab5 DN cells.

(Bottom-Right) During stretch application; WT junctions exhibit an average straightness of 0.96 ± 0.05, and Rab5DN junctions show a straightness of 0.87 ± 0.11. In both WT and Rab5DN cells, the application of stretch increased junctional straightness. The straightness of Rab5DN junctions increased to values similar to those of WT before stretch application, however they continued to exhibit much lower straightness than the stretched WT cells. N_{WT}=224 junctions on 5 embryos and N_{Rab5DN}=279 junctions on 5 embryos.

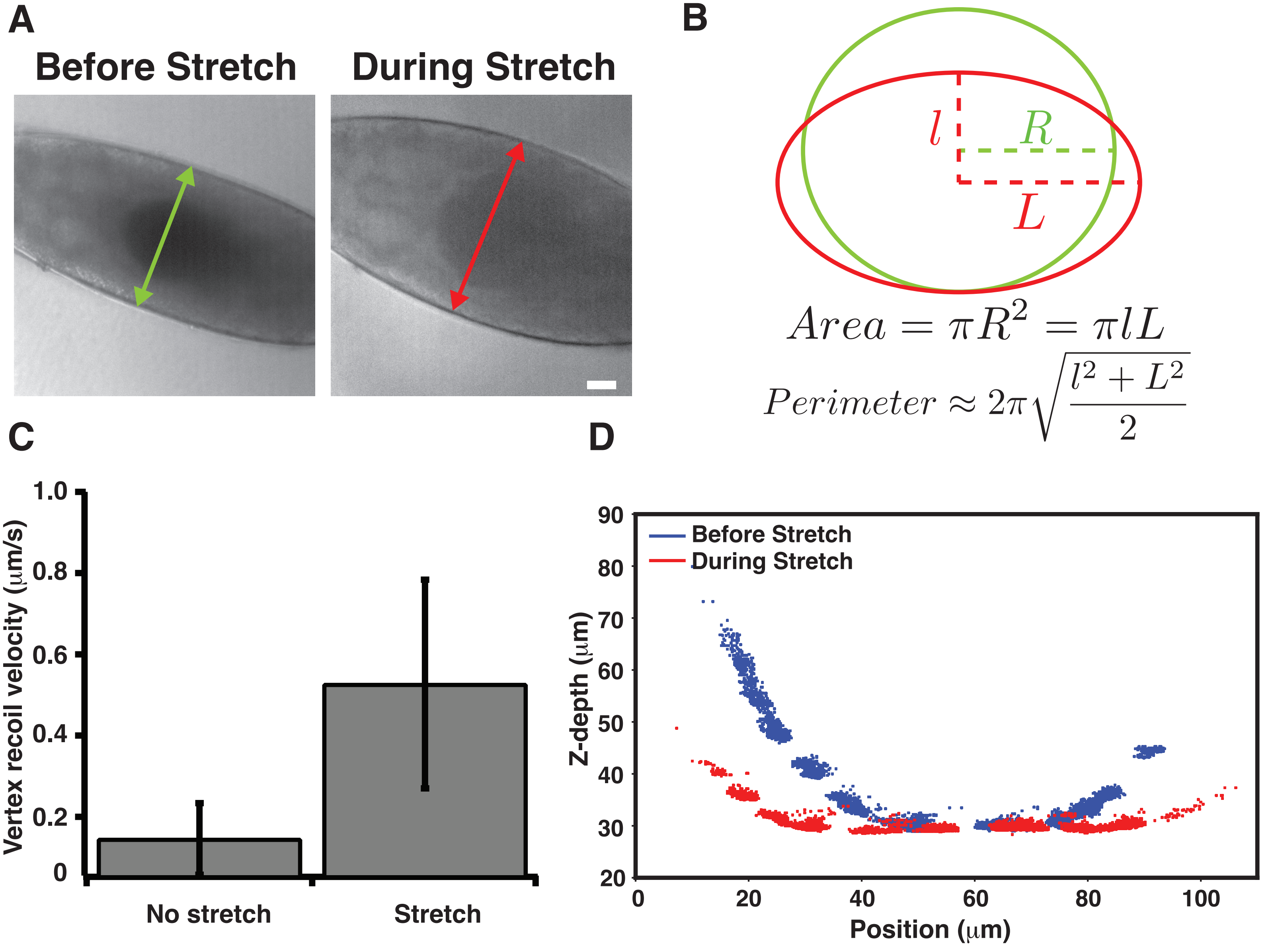

A
Before Stretch
During Stretch
B
l
R
L
$Area = \pi R^2 = \pi l L$
$Perimeter \approx 2\pi\sqrt{\frac{l^2 + L^2}{2}}$
C
Vertex recoil velocity (μm/s)
1.0
0.8
0.6
0.4
0.2
0
No stretch
Stretch
D
Before Stretch
During Stretch
Z-depth (μm)
90
80
70
60
50
40
30
20
0
20
40
60
80
100
Position (μm)

Figure S3. Embryonic deformations and stretch generated by squeezing embryos (related to figure 2)

(A) Example of an embryo imaged by transmission light before and during stretch induction. We observe an increase in width, characteristic of a change in aspect ratio. Scale bar: 40µm.

(B) Schematics showing a typical change in aspect ratio and the estimation of the change in height for a measured change in embryo width. Assuming the area of the central region of the embryo is conserved upon stretch, we calculated, from the change in embryo width, a change in height of approximately 25 % in our experiments. From this change in aspect ratio, we calculated a 8% increase in perimeter.

(C) Average vertex recoil velocity after laser dissection of WT junctions in the case where no stretch had been applied and with stretch applied. We observe an increased initial recoil velocity. $N_{NoStretch}$=22 junctions and $N_{Stretch}$=7 junctions. Error bars are standard deviations. Note that the low number in the case of stretched junctions is due to to the complexity of the experiments as embryos tend to "blast" when dissection is applied during stretch.

(D) Profiles of the central region of the embryo along the AP axis before (blue) and during (red) stretch application. We observe a change in aspect ratio from a profile nearly circular to nearly elliptic.

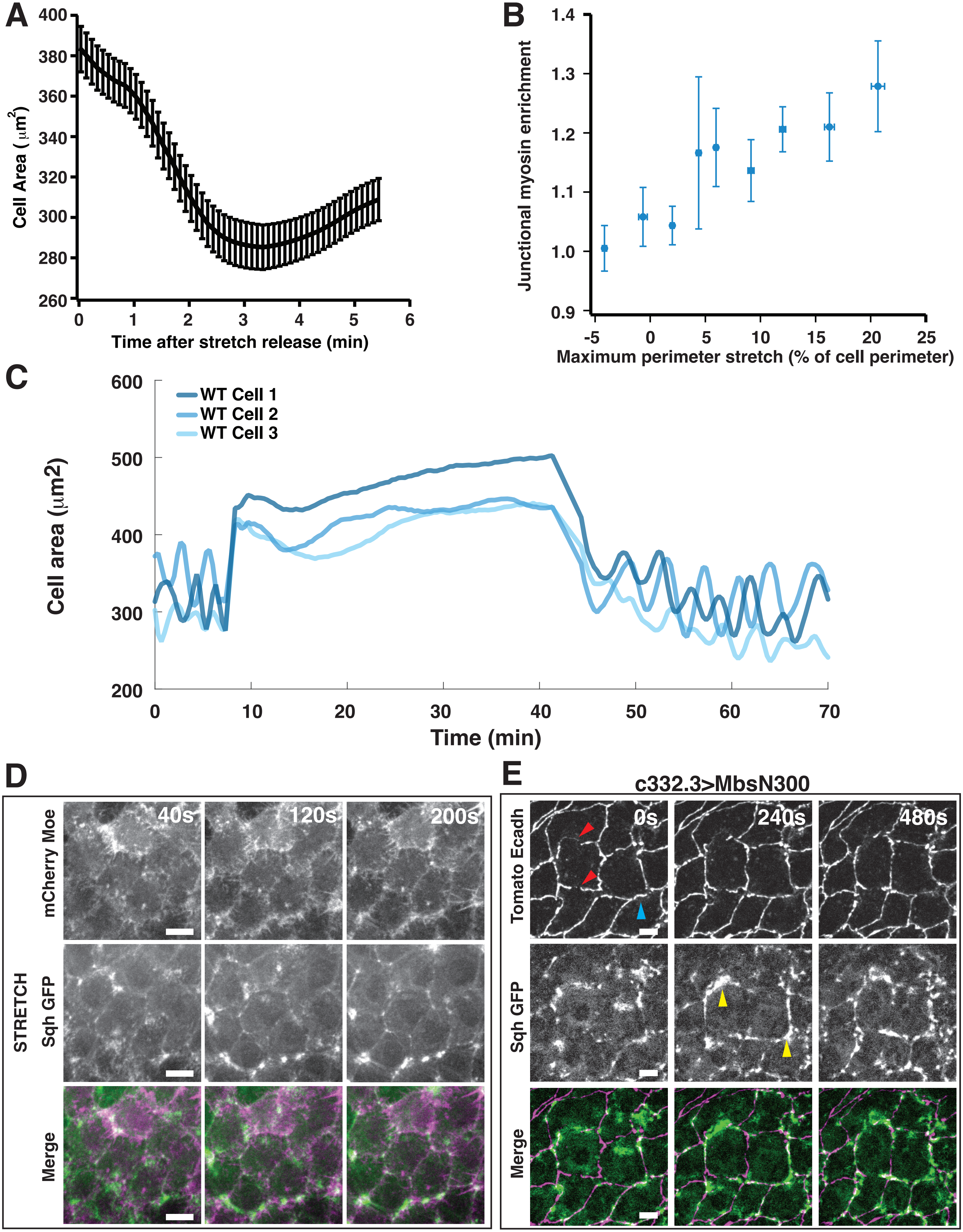
A
Cell Area (μm2)
Time after stretch release (min)
B
Junctional myosin enrichment
Maximum perimeter stretch (% of cell perimeter)
C
WT Cell 1
WT Cell 2
WT Cell 3
Cell area (μm2)
Time (min)
D
STRETCH
mCherry Moe
Sqh GFP
Merge
40s
120s
200s
E
c332.3>MbsN300
Tomato Ecadh
Sqh GFP
Merge
0s
240s
480s

Figure S4. Cellular response to ectopic stretch (related to figure 2)

(A) Average cell area over time after stress release. We observe a decrease of the area of approximately $100\mu m^2$ within the first 3 minutes. N=61 cells on 6 embryos. Error bars show standard errors.

(B) Graph showing the enrichment in myosin (defined as the ratio of the peak in myosin concentration at junctions by the initial junctional concentration) as a function of the perimeter stretch (defined as the ratio of the maximum perimeter after stretch by the average perimeter before stretch). We observe a linear correlation between the myosin enrichment and the perimeter stretch. Error bars show standard errors. N=88 cells on 7 embryos.

(C) Graph showing the apical cell surface area as a function of time for 3 individual cells before stretch, during stretch and after release. The three cells are the same as those shown in Fig2-B.

(D) Time lapse sequence showing the localization of actin (labelled with mCherry Moe) and myosin (Sqh-GFP) during stretch. We observe a progressive localization of both actin and myosin at junctions. Note that the expression of mCherry-Moe is patchy with some cells displaying lower expression due to the Gal4 driver. Scale bar: $10\mu m$.

(E) Time lapse sequence showing AS cells expressing *DE Cad-Tomato, Sqh-GFP*, *C332.3 Gal4/UAS MbsN300*. Stable gaps in adherens junctions can be observed consequent to the lower activity of myosin (Red arrowheads). Progressive disassembly of junctions can also be observed (blue arrowheads). We observe that myosin localizes to the juntional gaps but is unable to restore junctional integrity (yellow arrowheads). Scale bar: 10μm.

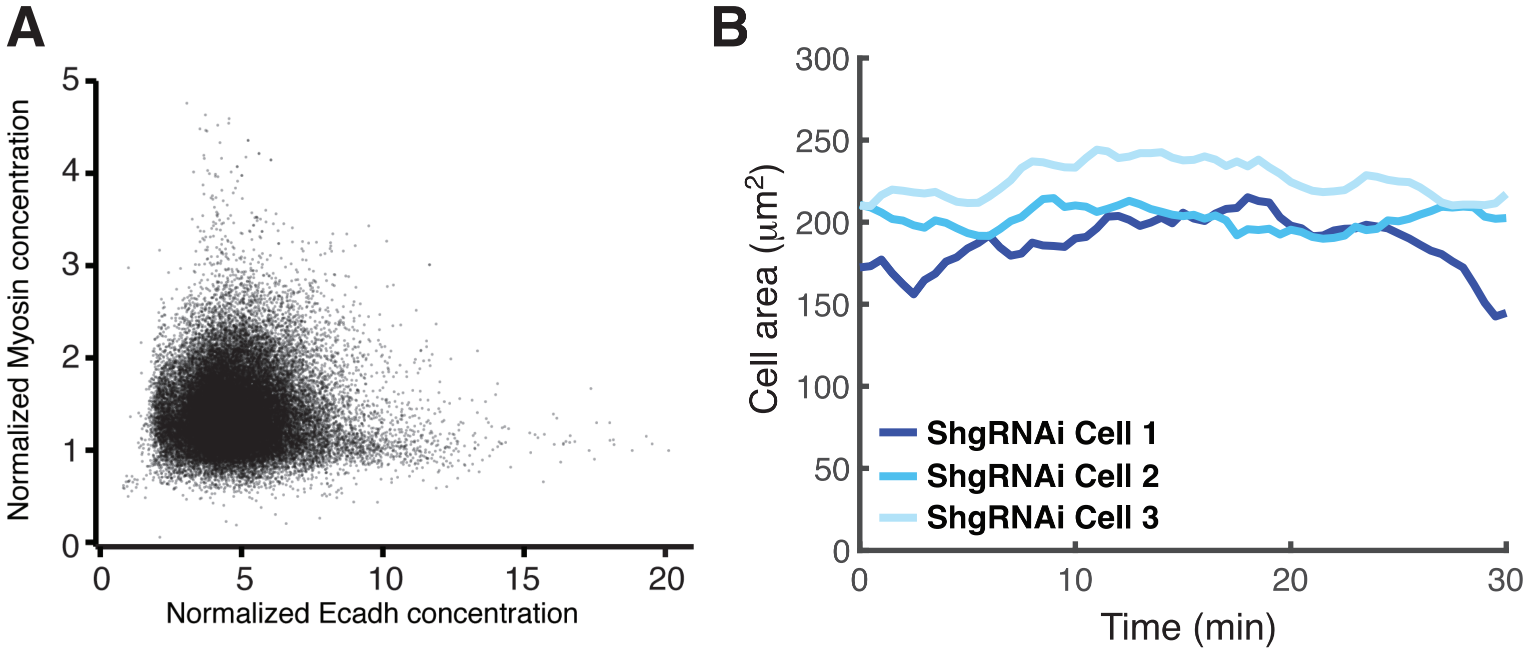
A
Normalized Myosin concentration
Normalized Ecadh concentration
0
1
2
3
4
5
0
5
10
15
20
B
Cell area (μm²)
Time (min)
300
250
200
150
100
50
0
0
10
20
30
ShgRNAi Cell 1
ShgRNAi Cell 2
ShgRNAi Cell 3

Figure S5 Adherens junctions and pulsatile activity of the AS cells during DC in WT and Shg-RNAi (related to figure 4).

(A) Graph showing the raw data that has been binned to produce the graph in Figure 4-B. Individual cells over time for 5 minutes using the SAC analysis. From each individual SAC, junctional pixels were selected, as those less than 1.5 pixels from the border, and their myosin and E-cadherin intensities (normalized by the initial value at the cell centre) were plotted against each other. Each dot therefore represents a single junctional pixel from an individual scaled average cell. We observe that pixels with the highest E-cadherin levels have the lowest myosin levels and conversely pixels with the highest myosin levels have low E-cadherin levels.

(B) Graph showing cell area over time for three individual AS cells with downregulated E-cadherin (expressing Shg-RNAi). We observe a steady area and an absence of pulsatile activity.

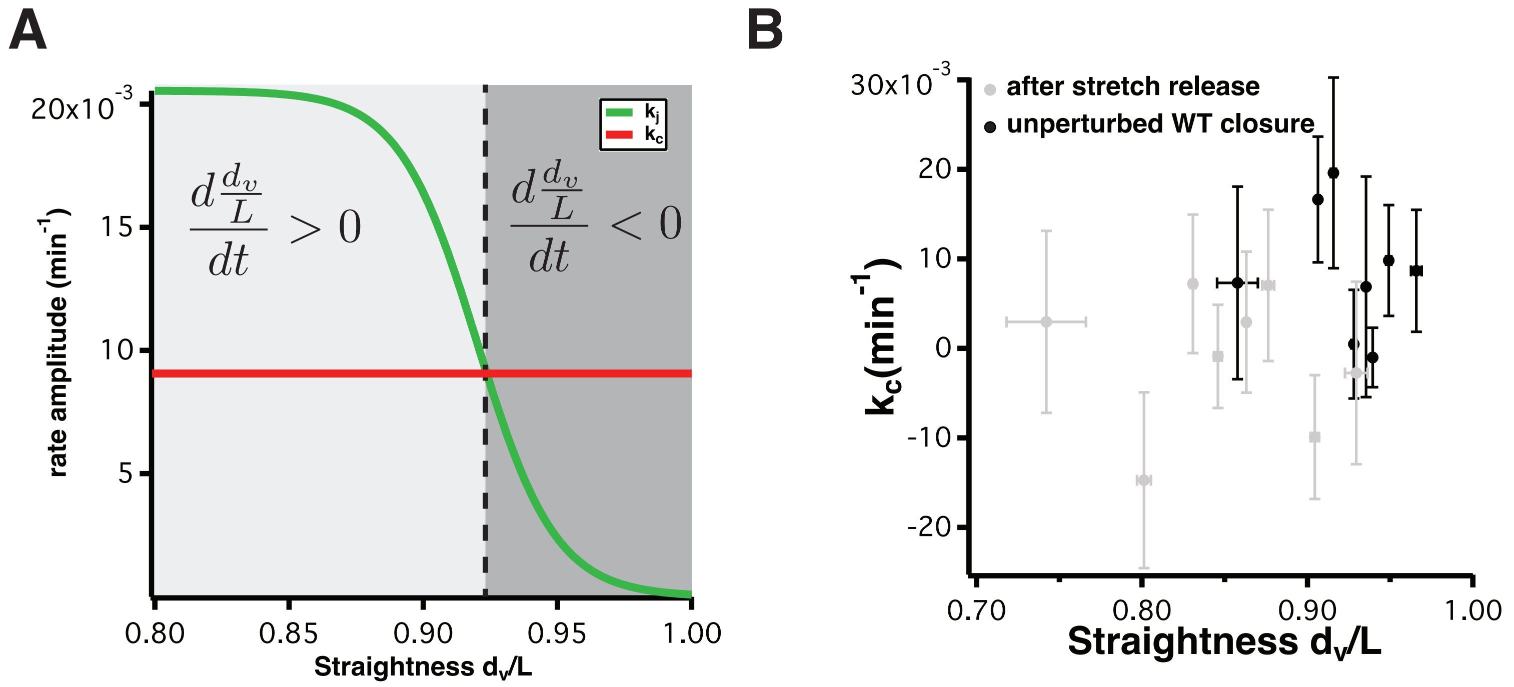

A
20x10-3
15
10
5
rate amplitude (min-1)
kj
kc
$\frac{d\frac{d_v}{L}}{dt} > 0$
$\frac{d\frac{d_v}{L}}{dt} < 0$
0.80
0.85
0.90
0.95
1.00
Straightness dv/L
B
30x10-3
20
10
0
-10
-20
kc(min-1)
after stretch release
unperturbed WT closure
0.70
0.80
0.90
1.00
Straightness dv/L

Figure S6. Straightness maintenance and rate dependencies (related to figure 6).

(A) Graph showing the fitted curve to the rate k_j as a function of the straightness, and the average value of k_c during unperturbed closure. We define two regions of low and high straightness separated by the intersection of the two curves. For low straightness, $k_j > k_c$, inducing an increase of the junction straightness, which brings the straightness back to the intersection of the two curves. For high straightness, $k_c > k_j$, inducing a reduction in straightness towards the intersection.

(B) Graph showing the contraction rate $k_c$as a function of the straightness for AS cell adherens junctions during WT DC (Black) and after stretch release (Gray). There is no clear dependency of $k_c$with straightness in the case of unperturbed closure. N=50 junctions on 3 embryos, error bars show standard errors.
