# Supplementary material for: Adherens Junction Length during Tissue Contraction Is Controlled by the Mechanosensitive Activity of Actomyosin and Junctional Recycling
Source: Dev Cell. 2018 Nov 19;47(4):453–463.e3. doi: 10.1016/j.devcel.2018.10.025 (PMC6291457; doi:10.1016/j.devcel.2018.10.025)
Supplement: Document S2. Article plus Supplemental Information [file mmc11.pdf]

Article

Developmental Cell

Adherens Junction Length during Tissue Contraction Is Controlled by the Mechanosensitive Activity of Actomyosin and Junctional Recycling

Graphical Abstract

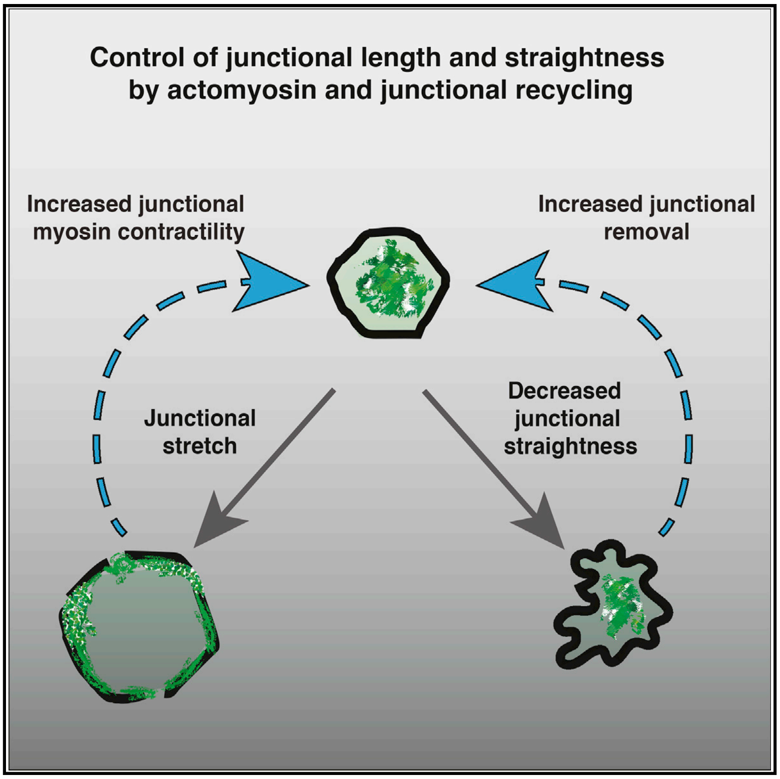

Authors

Angughali Sumi, Peran Hayes, Arturo D'Angelo, Julien Colombelli, Guillaume Salbreux, Kai Dierkes, Jérôme Solon

Correspondence

dierkes.kai@googlemail.com (K.D.),
jerome.solon@crg.es (J.S.)

In Brief

The control of adherens junction length is essential for tissue contraction. Sumi et al. show that junctional length is controlled by actomyosin and junctional recycling. When junctions are stretched, actomyosin relocalizes to junctions to reduce their length. When junctions are ruffled, junctional removal is enhanced to restore high junction straightness.

Highlights

- Adherens junction length is controlled by actomyosin and junctional recycling
- Upon stretch, actomyosin is enriched at junctions, and contractile pulses are arrested
- Excess junctional length enhances junctional removal, restoring high straightness
- E-cadherin levels control cell actomyosin distribution and contractile pulses

Sumi et al., 2018, Developmental Cell *47*, 453–463
November 19, 2018 © 2018 Elsevier Inc.
https://doi.org/10.1016/j.devcel.2018.10.025

CellPress

Developmental Cell
Article

CellPress

Adherens Junction Length during Tissue Contraction Is Controlled by the Mechanosensitive Activity of Actomyosin and Junctional Recycling

Angughali Sumi,[1,5] Peran Hayes,[1,5] Arturo D'Angelo,[1] Julien Colombelli,[3] Guillaume Salbreux,[4] Kai Dierkes,[1,*] and Jérôme Solon[1,2,6,*]
[1]Centre for Genomic Regulation (CRG), The Barcelona Institute of Science and Technology, Dr. Aiguader, 88, Barcelona 08003, Spain
[2]Universitat Pompeu Fabra (UPF), Barcelona 08003, Spain
[3]Institute for Research in Biomedicine (IRB Barcelona), The Barcelona Institute of Science and Technology, Barcelona 08028, Spain
[4]Francis Crick Institute, 1 Midland Road, London NW1 1AT, UK
[5]These authors contributed equally
[6]Lead Contact
*Correspondence: dierkes.kai@googlemail.com (K.D.), jerome.solon@crg.es (J.S.)
https://doi.org/10.1016/j.devcel.2018.10.025

SUMMARY

During epithelial contraction, cells generate forces to constrict their surface and, concurrently, fine-tune the length of their adherens junctions to ensure force transmission. While many studies have focused on understanding force generation, little is known on how junctional length is controlled. Here, we show that, during amnioserosa contraction in *Drosophila* dorsal closure, adherens junctions reduce their length in coordination with the shrinkage of apical cell area, maintaining a nearly constant junctional straightness. We reveal that junctional straightness and integrity depend on the endocytic machinery and on the mechanosensitive activity of the actomyosin cytoskeleton. On one hand, upon junctional stretch and decrease in E-cadherin density, actomyosin relocalizes from the medial area to the junctions, thus maintaining junctional integrity. On the other hand, when junctions have excess material and ruffles, junction removal is enhanced, and high junctional straightness and tension are restored. These two mechanisms control junctional length and integrity during morphogenesis.

INTRODUCTION

The contraction of epithelia is an essential mode of tissue remodeling occurring during several development processes, such as gastrulation or neural tube closure in humans (Llimargas and Casanova, 2010; Sawyer et al., 2010). It consists of a collective cell shape change generating a major reduction in tissue surface and eventually leading to an epithelial invagination. During this process, cells have to generate forces to constrict their own surface and at the same time fine-tune the length of their adherens junctions to ensure the epithelial homeostasis necessary for force transmission (Lecuit et al., 2011; Niessen et al., 2011). Contractile forces are usually generated by the activity of the actomyosin cytoskeleton: actomyosin localizes preferentially at the apical site of the cells, generating contractile forces pulling on the neighboring cells and consequently reducing the apical surface (Lecuit et al., 2011; Martin and Goldstein, 2014). While many studies have described the mechanisms at the origin of force generation during tissue contraction in various developmental processes, much less is known on how cell-cell junction remodeling and epithelial homoeostasis is ensured during this process. To understand how adherens junctions remodel during epithelial contraction, we used *Drosophila* dorsal closure as a model system.

Drosophila dorsal closure (DC) is a classical model to investigate epithelial contraction during development (Jacinto et al., 2002). It consists of the closure of an epidermal gap on the dorsal side of the embryo and relies, among other forces, on the contraction of the amnioserosa (AS) tissue (Hayes and Solon, 2017; Hutson et al., 2003; Kiehart et al., 2000). In this process, the contraction is associated with a reduction in individual AS cell volume triggered by the activation of the apoptotic program (Saias et al., 2015). Together with volume reduction, AS cells display actomyosin-based contractile pulses that periodically stretch and crumple adherens junctions (Gorfinkiel et al., 2009; Hara et al., 2016; Solon et al., 2009). However, adherens junctions also decrease in length during the contraction, thus maintaining a global homeostasis of the tissue and a constant epithelial tension (Saias et al., 2015). The mechanisms controlling junctional length and tension during DC are still unknown.

Here, we uncover mechanisms controlling adherens junction length and integrity during DC. We provide evidence that junctional length and integrity are maintained due to the combination of the mechanosensitive activity of the actomyosin cytoskeleton and of a straightness-dependent junction removal mechanism. Our results show that the actomyosin cytoskeleton can relocate from the medial area of cells, i.e., from the actomyosin cortex at the center of the apical cell surface, to the surrounding adherens junctions upon external stress application. This relocation is associated with a switch in cell behavior from pulsatile to non-pulsatile. By modulating E-cadherin levels, we can generate

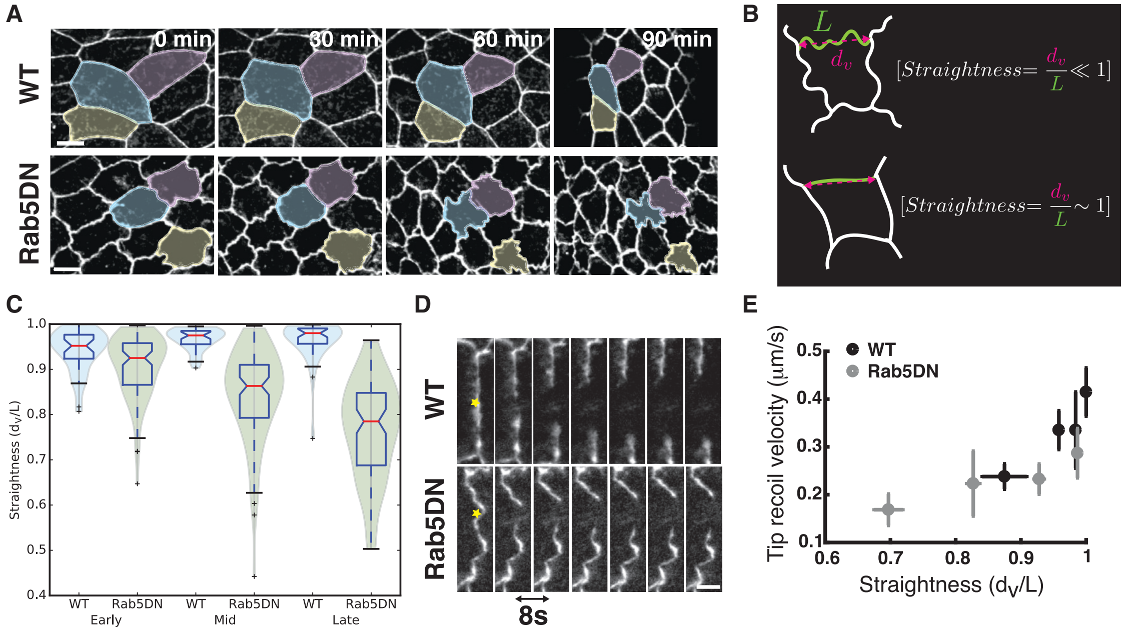

Figure 1. The Scaling of Junctional Length with Contraction Relies on Endocytosis

(A) Time-lapse images of AS cells expressing E-cadherin-GFP showing the decrease in their apical surface area (color shaded regions) during dorsal closure in the case of WT (top) and Rab5DN-expressing cells (bottom). While in both WT and Rab5DN embryos, the apical surface area decreases, WT cells are able to maintain straight junctions, whereas Rab5DN-expressing cells are unable to remove junctional material at the appropriate rate, resulting in ruffled junctions. Scale bar: 10 μm.

(B) Schematics presenting the definition of straightness. (Top) In the case of a ruffled junction, the junction path length of the junction L is larger than the inter-vertex distance d_v, resulting in a straightness smaller than 1. (Bottom) In the case of a straight junction, the junction path length and inter-vertex distance are similar, resulting in a straightness close to 1.

(C) Graph showing junctional straightness for WT (blue) and Rab5DN-expressing (green) embryos at early, mid, and late phases of DC. While the straightness is approximately constant in WT, it decays in Rab5DN-expressing cells. For WT embryos, late DC is defined as when the width of the opening reaches 50 microns, and mid and early time points were taken at 57 and 111 min before this, respectively. In the case of Rab5DN, embryos do not complete DC and never reach an opening width of 50 microns. Therefore, late DC was considered as when closure arrests. N_{WT} = 35 junctions on 2 embryos and N_{Rab5DN} = 90 junctions on 2 embryos.

(D) Time lapses showing junction retraction kinetics after laser dissection in WT and Rab5DN-expressing cells. Retraction velocity is higher in WT junctions than in Rab5DN Junctions, indicating a higher tension. The time sequence interval is 8 s. Yellow star marks the location of the cut. Scale bar: 2 μm.

(E) Graph showing the average initial recoil velocity of the junction tips after laser dissection as a function of junction straightness for WT and Rab5DN-expressing cells. We observe an increase in junctional tension when reaching a straightness close to 1. Each point is an average of 5–6 junctions for WT and 10–11 junctions for Rab5DN. The corresponding raw data are presented in Figure S1B. Error bars show standard errors of the mean. N_{WT} = 22 junctions and N_{Rab5DN} = 43 junctions.

a similar change in actomyosin distribution, from medial to junctional. Concurrently, we also induce a switch in cell behavior, from pulsatile to non-pulsatile, identifying E-cadherin levels as a regulator of actomyosin contractility and cell dynamics within tissues. Combining a theoretical description with the experimental assessment of contraction and junction removal rates, we show that the rate of junction removal depends on the junctional straightness during closure and that this dependence is sufficient to generate a stable straightness during tissue contraction. Altogether, we find that junctional length is actively controlled during epithelial contraction. When junctions are ruffled, (1) actomyosin is located in the medial array, generating contractile pulses, and (2) excess junction is removed during tissue contraction in an endocytosis-dependent manner. When junctions are stretched, myosin relocalize to junctions, maintaining their integrity. Our results point toward E-cadherin as a regulator of the actomyosin distribution in the cell and thus cell dynamics.

RESULTS

Junctional Straightness Is Constant during Epithelial Contraction

In order to maintain the global tissue geometry, the decrease in AS cell area observed during DC must be coordinated with a decrease of the adherens junctions' length (Figure 1A). Using embryos expressing E-cadherin tagged with GFP, we observed that, during the 110 min of progression of closure, the average cell area decreased from 220 ± 50 μm^2 to 106 ± 40 μm^2, while the average inter-vertex distance and path length decreased from 12.3 ± 4.8 μm to 8.05 ± 2.9 μm and from 13.1 ± 5.0 μm to 8.2 ± 2.9 μm, respectively (Figure 1A). To study adherens junction geometry, we define junctional straightness as the ratio of the inter-vertex distance to the path length of the junction, with a value comprised between 0 and 1 (as defined in Figure 1B). We observed that while junction lengths decrease by more than 50%, their straightness remains nearly constant (approximately

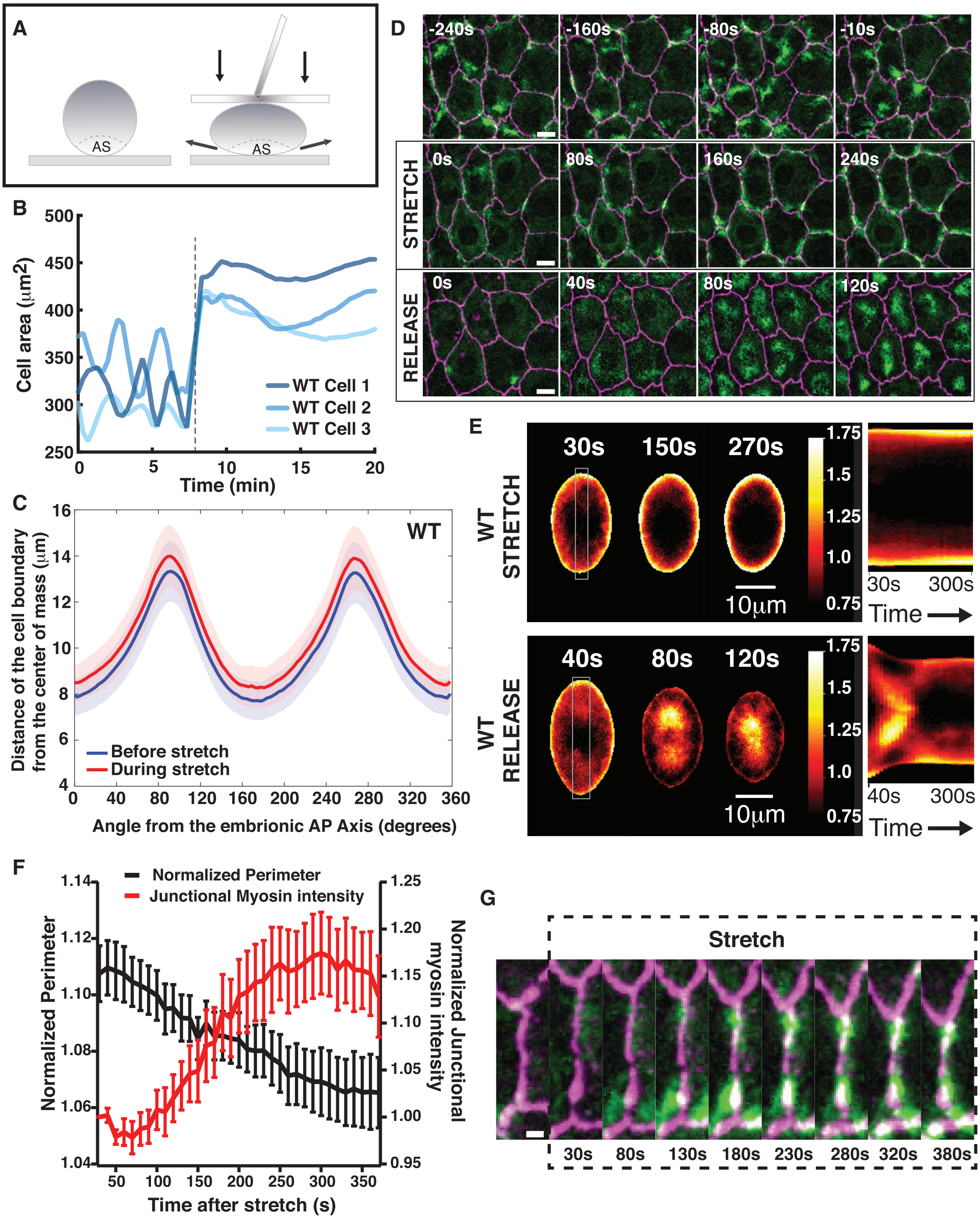

Figure 2. Response of AS Cells to Ectopic Mechanical Stretch

(A) Schematic showing the principle of the stretching experiments. Embryos are compressed using a coverslip. Changes in embryo aspect ratio lead to an increase in interface tension and a stretching of the AS tissue.

(B) Graph showing the apical cell surface area as a function of time for 3 individual cells before and during stretch. The onset of stretch is marked with a dashed line. Upon stretch, we observe an increase of the apical cell surface areas and an arrest of the oscillations.

(C) Graph showing the average distance between the cell boundary and the center of mass (extracted during the scaled average cell methodology) as a function of the angle from the anterior-posterior (AP) axis for average cells before (blue) and during stretch (Red). Upon stretch, we observe a homogeneous increase in centroid-boundary distance that is independent of the angle. Error bars show standard deviations. n = 58 cells on 5 embryos.

(D) Time-lapse sequence showing AS cell kinetics of embryos expressing Tomato-E-cadherin and Sqh-GFP before, during, and after application of stretch. (Top) Before stretch, AS cells display myosin foci propagating through the medial area and a consequent pulsatile activity. (Middle) During stretch, AS cell apical surface area is approximately constant, the myosin foci are not present anymore, and myosin localizes preferentially at junctions. (Bottom) Immediately

(legend continued on next page)

0.95) (Figures 1A and 1C; Video S1). This indicates a direct scaling of adherens junction length with the contraction of the tissue and opens the question of how such scaling is controlled. To decrease junctional length, excess junctional material must be removed. A natural candidate for junctional material removal is the endocytic machinery (Levayer et al., 2011; Mateus et al., 2011). To explore the role of endocytosis on junction lengths, we impaired endocytosis by expressing a dominant negative form of Rab5 (Rab5DN) specifically in the AS tissue. Rab5 is a small GTPase associated with early endosomes that is involved in the regulation of the endocytic rate (Bucci et al., 1992). At early stages of DC, before the contraction of the AS starts, we observed that the junctional straightness of Rab5DN cells is similar to wild-type (WT) cells (approximately 0.9). As contraction progresses, the inter-vertex distance reduces faster than the junctional length, leading to ruffled junctions and a slower closure (Figures 1A and 1C; Video S1). Endocytosis is therefore required to adjust the reduction in junctional length to the rate of contraction of the tissue.

This begs the question: how is junctional length controlled? In other words, how do cells make sure that their junctions are not overstretched (leading to compromised junction integrity) or ruffled during epithelial contraction?

A way to prevent junction overstretching would be to increase junctional tension when straightness reaches values close to 1. A relationship between junctional straightness and tension has already been identified in the context of pulsed junctional contractions (Hara et al., 2016). To investigate whether tension increases when reaching high straightness, we used laser dissection to cut individual junctions in WT and Rab5DN cells (Figure 1D and Video S2). By measuring the initial relaxation velocity after a cut, we can estimate relative junctional tensions, under the assumption that friction is constant between junctions (Colombelli and Solon, 2013; Farhadifar et al., 2007; Rauzi et al., 2008). We observed that for WT and Rab5DN-expressing cells, junctional tension varies with straightness; the initial retraction velocity being around 0.2 μm/s for low straightness values and increasing up to 0.4 μm/s when straightness approaches 1 (Figures 1E and S1A–S1C). This suggests that maintaining high junctional straightness during contraction allows the junctional tension to remain high. In addition, because, at high straightness, small changes in straightness generate large changes in tension, this results in a higher tension sensitivity (Figure 1E). In this regime, a small increase in straightness would induce a large increase in tension, which could in turn prevent overstretching.

Method to Ectopically Stretch AS Cells: Squeezing *Drosophila* Embryos

To explore the mechanism by which cells prevent overstretching, we developed a method to ectopically stretch the AS tissue using an external force (see STAR Methods and Figure 2A). By squeezing embryos between two coverslips, we were able to induce a strain in the center of the embryo along the lateral direction of approximately 30% (Figures S3A and S3D). Assuming the central section of the embryo being transformed was similar to an ellipse of constant area, we could estimate a reduction in embryo height of approximately 25%, resulting in a perimeter increase of the central section of the embryo of approximately 8% (Figure S3B). This change in transversal aspect ratio of the embryo consequently induces a stretch of the AS cells. Using embryos expressing a tagged version of myosin II (Sqh-GFP) and Tomato E-cadherin, we could segment the AS cells over time and estimate the changes in area and perimeter (Figures 2B and S1D). Upon stretch, we found an average increase of 8.4% ± 12% in cell area and 3% ± 6.4% in perimeter (Figures S2A and S2B). To further characterize our stretching experiments and quantify the changes in cell shape during stretch, we generated a scaled average cell (SAC) by averaging cell shape over many cells at each time point (see STAR Methods and Figure S1E). In this way, we could quantify the anisotropy of stretch during our stretching experiment along the anterior-posterior (AP) and dorsal-ventral (DV) axis. We found that the stretch is close to being isotropic with a 6%–8% change along all radial directions (Figures 2C and 3C). Altogether, our results show that our squeezing method is generating a fast stretch of the apical surface of the cells, allowing us to monitor cellular response to mechanical stretch.

AS Cell Stretch Induces the Relocalization of Actomyosin to Junctions and Arrests Contractile Pulses

To monitor how the actomyosin cytoskeleton reacts to ectopic stretch, we used embryos expressing myosin II (Sqh-GFP) and Tomato E-cadherin. We observed that, upon stretch, cell pulses arrested and cells maintained an approximately constant area over a period of a few tens of minutes (Figures 2B and 2D; Video S3). Concurrently, the myosin foci that were previously present in the center of the apical cell surface (medial area) disappeared, and punctuated myosin enrichment formed at the junctions (Figure 2D). After release of the stretch, together with a decrease in cell surface area across the entire AS tissue, we observed a relocalization of myosin from the junctions to the center of the cells (Figures 2D and S4A; Video S4). Pulsed

after stress release, myosin flows from the junctions to the medial area in a coordinated manner, generating a concerted decrease in cell area. Scale bar: 10 μm.

(E) (Left) Scaled average cells over time for WT cells during stretch (Top-Left) and after release (Bottom-Left); and (Right) corresponding kymographs showing myosin distribution within the gray box (averaged horizontally) over time. We observe a progressive enrichment at the junction with time under stretch and flows from the junction to the middle of the cell after stress release. $N_{WTstretch}$ = 58 cells on 5 embryos and $N_{WTrelease}$ = 61 cells on 6 embryos.

(F) Graph showing the normalized junctional myosin concentration and normalized perimeter over time during stretch for cells experiencing a stretch larger than 9%. Junctional myosin is defined as myosin intensity measured within a 3-pixel width path following the E-cadherin-stained junction. Myosin levels are normalized to the myosin levels at the onset of the stretch. Perimeter is normalized by the perimeter before stretch. Error bars show standard deviations. N = 14 cells on 4 embryos.

(G) Time sequence showing an adherens junction experiencing ectopic stretch in an embryo expressing Tomato-E-cadherin and Sqh-GFP. After stretch, myosin levels at the junction increase, and junctional length reduces to approximately similar length than before stretch was applied. Scale bar: 2 μm.

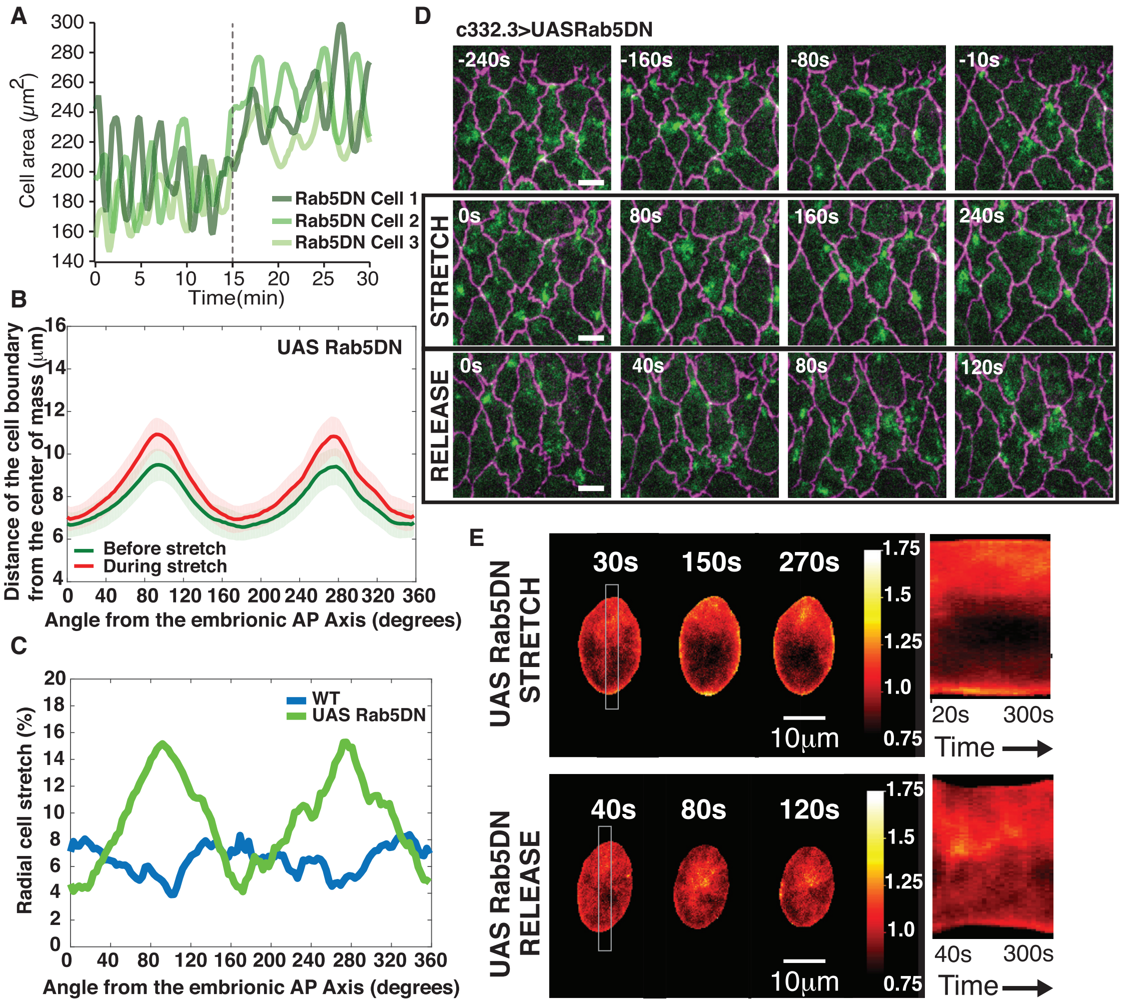

Figure 3. Response of Rab5DN-Expressing AS Cells to Ectopic Stretch

(A) Graph showing the apical cell surface area as a function of time for 3 individual cells in Rab5DN-expressing embryos before and during stretch. The onset of stretch is marked with a dashed line. Upon stretch, we observe an increase in the apical cell surface areas without any arrest of the area oscillations.

(B) Graph showing the average distance between the cell boundary and the center of mass (extracted during the scaled average cell methodology) as a function of the angle from the anterior-posterior (AP) axis for average cells before (green) and during (red) stretch for Rab5DN expressing-cells. We observe a larger increase of the centroid-border distance along the lateral directions (90 and 270 degrees). Error bars show standard deviations. n = 70 cells on 5 embryos.

(C) Proportion of stretch as a function of the angle from the anterior-posterior axis for WT and Rab5DN-expressing cells (extracted from Figures 2C and 3B, respectively). We observe a relatively isotropic stretch of 6%–8% for the WT and an anisotropic stretch, reaching 15% along the lateral direction, for Rab5DN-expressing embryos.

(D) Time-lapse sequence showing AS cell kinetics of embryos expressing *DE Cad-Tomato, Sqh-GFP*, and *C332.3 Gal4/UAS Rab5DN* before, during, and after application of stretch. (Top) Before stretch, AS cells display myosin foci propagating through the medial area, a consequent pulsatile activity, and excess junctional material in the form of ruffles. (Middle) During stretch, AS cell apical surface area is increased, but unlike WT cells, the junctions continue to exhibit ruffles, the myosin foci are still present, and cells remain pulsatile. (Bottom) After stretch release, myosin continues to pulsate in a manner similar to that prior to the stretch application.

Note that, while WT and Rab5DN perimeters are similar, the area of Rab5DN cells is on average smaller than in WT. This arises from the experimental methodology: we select the most dorsal AS cells for stretching, which are the largest in WT embryos, while such inhomogeneity in cell size is less present in Rab5DN cells. In addition, Rab5DN cells were selected to exhibit ruffles and therefore may be at slightly later stages of closure. Scale bar: 10 μm.

(E) (Left) Scaled average cells over time for Rab5DN cells during stretch (Top-Left) and after release (Bottom-Left); and (Right) corresponding kymographs showing myosin distribution within the gray box (averaged horizontally) over time. In contrast with WT, we do not observe any significant enrichment at the junctions with time under stretch and only a slight myosin relocation from the junction to the middle of the cell after stress release. $N_{Rab5DNstretch}$ = 70 cells on 5 embryos and $N_{Rab5DNrelease}$ = 70 cells on 5 embryos.

contractions reappeared with an initial synchronous pulse of all previously stretched cells. After this pulse, AS cells continued oscillating in an asynchronous manner with myosin foci flowing through the medial array, in a similar fashion to before the stress was applied (Figure S4C and Video S4). Extending our SAC methodology to average myosin spatial distribution over many cells at each time point, we generated an average profile of the apical AS cell surface during and after the application of

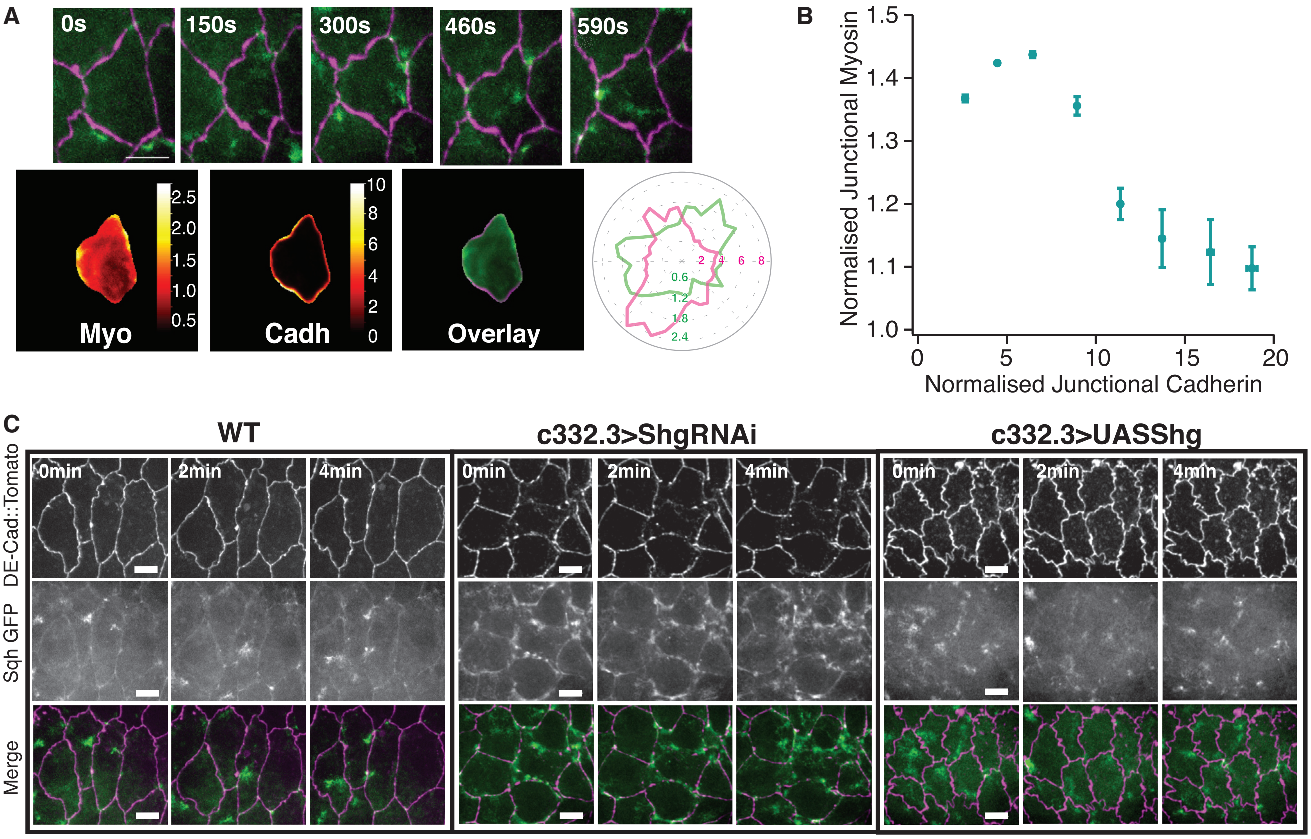

Figure 4. Modulations in E-Cadherin Impact Junction Straightness and Myosin Localization

(A) (Top) Time-lapse imaging showing an AS cell expressing Tomato E-cadherin and Sqh-GFP. Myosin II localizes transiently at discontinuities in junctional E-cadherin concentration. Scale bar: 10 μm. (Bottom) Scaled average cell analysis of the above cell, averaged over a period of 10 min for myosin, E-cadherin, and the overlay. On the right, a radial plot displays the amplitude of the levels of myosin (green) and E-cadherin (magenta) at the scaled average cell boundaries for each angle. Overall, myosin enrichments at the junction correlate with regions of low E-cadherin levels.

(B) Graph showing the levels of normalized junctional myosin II (normalized by the initial value at the cell center) as a function of the normalized junctional E-cadherin for boundary pixels ($\leq$1.5 pixels from the cell edge) of all individual scaled average cells. We observe a global anti-correlation, with high levels of myosin for low E-cadherin levels and vice versa. n = 58 cells on 5 embryos. Error bars show standard errors.

(C) (Left) Time lapse of AS cells expressing *DE Cad-Tomato, Sqh-GFP*. Cells are pulsatile, and myosin is localized in foci within the cells and also at junctions. Scale bar: 10 μm. (Middle) Time lapse of AS cells expressing *DE Cad-Tomato, Sqh-GFP*, and *C332.3 Gal4/UAS ShgRNAi*. Cell pulsation is impaired, and myosin is preferentially localized at junctions. Scale bar: 10 μm. (Right) Time lapse of AS cells expressing *DE Cad-Tomato, Sqh-GFP*, and *C332.3 Gal4/UAS Shg*. Adherens junctions are ruffled, and myosin is preferentially localized in the medial array. Scale bar: 10 μm.

stretch (see STAR Methods; Figures S1E and 2E). During stretch, we observed a progressive increase in myosin levels at the junctions and a reduction in the central part of the cell (Figures 2D and 2E; Video S5). The level of this junctional myosin enrichment is proportional to the extent to which the cell perimeter has been stretched (Figure S4B). For the most stretched cells (>9% perimeter stretch), the enrichment in myosin occurred concurrently with reduction in junctional length and overall perimeter of a few percents, suggesting the myosin II relocation and contraction as a mechanism to maintain junctional length upon stretch (Figures 2F and 2G). Conversely, when stretch was released, we observed a flow of myosin from the junction to the cell center, resulting in a decrease in myosin levels at the adherens junction and an increase in the medial array (Figures 2D and 2E; Video S6). Similar changes are observed when imaging the actin cytoskeleton (Figure S4D).

Excess Junctional Material Prevents Both the Actomyosin Relocation and Pulse Arrest upon Stretch

To investigate whether the stretch experienced by the adherens junctions is at the origin of the change in actomyosin distribution, we performed the same stretching experiment on cells expressing Rab5DN. In these conditions, adherens junctions exhibited an excess length in the form of ruffles. We therefore expected that this excess length would buffer the action of the stress application on the junctional straightness and consequently on tension. Using our methodology resulted in an overall larger and more anisotropic stretch than observed in WT, with larger deformation laterally than along the AP axis (Figures 3B and 3C). This increase in stretch compared to WT is possibly originating from a lower surface tension of the AS tissue due to the excess junctional material, therefore allowing more deformation for the same stress application. While the cell perimeter increased in Rab5DN embryos more than in WT embryos as a response to

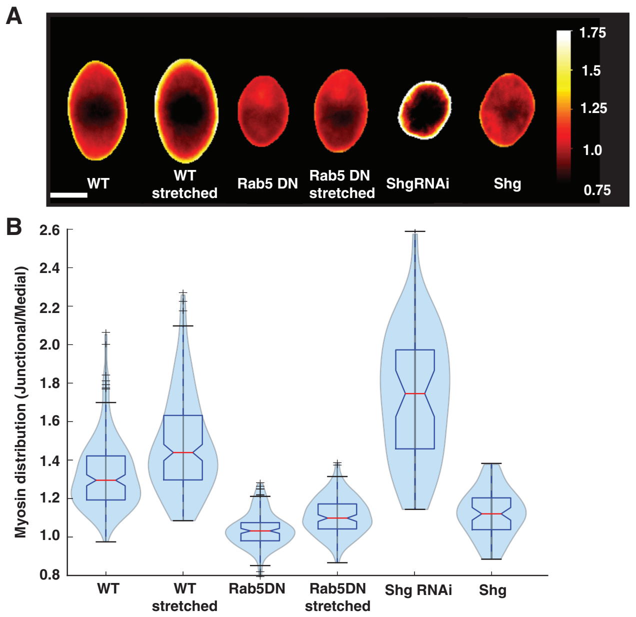

Figure 5. Myosin Distribution in AS Cells Presenting Different Levels of Stretch or E-Cadherin

(A) Scaled average cells showing the distribution of myosin in the case of WT, WT stretched, Rab5 DN, Rab5 DN stretched, and E-cadherin downregulation and overexpression (Shg RNAi and Shg overexpression, respectively). Myosin preferentially localizes to junctions when cells are stretched or E-cadherin levels are low.

(B) Histograms, corresponding to the average cells shown in (A), depicting the ratio of junctional myosin to medial myosin. Medial is defined as being further than 3 pixels from the segmented junction. Overall, myosin preferentially localizes to junctions when cells are stretched and when E cadherin is downregulated. (N_{WT} = 116 cells on 10 embryos, N_{Rab5DN} = 131 cells on 10 embryos, $N_{ShgRNAi}$ = 45 cells on 4 embryos, and N_{Shg} = 62 cells on 5 embryos).

stress application, junctions did not reach straightness values as high as observed in stretched WT embryos (Figures S2A and S2B). Under these stretch conditions, the Rab5DN cells did not stop pulsating, and although there was some minimal junctional enrichment of myosin, the foci continued to flow within the medial array (Figures 3A, 3D, and 3E; Videos S5 and S7) as if stretch had not been applied. This indicates that reaching high junctional straightness is essential to trigger actomyosin relocalization and pulse arrest. After release of the stretch, we also did not observe a synchronized flow of myosin from the adherens junctions to the cell center, as observed in WT conditions (Figures 2D and 3D). Using our SAC analysis, we could detect a slight relocalization of myosin from the junctions to the center in Rab5DN, consistent with a smaller relocation of myosin to junctions upon stretch in Rab5DN conditions, rather than a complete inhibition (Figure 3E).

Overall, our data show that AS cells display two distinct responses depending on the amount of mechanical stress: (1) upon low external stress, cells are pulsatile and actomyosin flows in the medial array, and (2) under high stress, myosin locates to adherens junctions, arresting the pulses. We propose that this mechanical response can act as a mechanism to prevent junction overstretching. This possibility is supported by the observation of stable junctional gaps and junction disruption in AS cells expressing MbsN300, an active form of the myosin phosphatase, which reduces the activity of myosin II in the tissue (Figure S4E).

Cellular Actomyosin Distribution and Pulsatile Activity Depend on E-Cadherin Levels at the Adherens Junctions

How does myosin sense junctional stretch? In the context of cell division, myosin can flow toward specific E-cadherin-depleted regions to promote the onset of cytokinesis (Pinheiro et al., 2017). To investigate a potential mechanosensitive role of E-cadherin in the context of epithelial contraction, we analyzed its interplay with myosin. During DC, while the cell surface area oscillates, we observed that myosin regularly accumulates at specific junction locations (Figure 4A). To quantify patterns of accumulation within each individual cell, we used our SAC methodology on a single cell, averaged over time. We observed that regions enriched in E-cadherin are often depleted in myosin, and vice versa (Figure 4A). This apparent anti-correlation observed on an individual cell is confirmed by plotting the E-cadherin and myosin junctional levels of n = 58 cells (Figures 4B and S5A).

We then decided to down- and upregulate E-cadherin levels specifically in the AS tissue to investigate the impact of E-cadherin on myosin localization. In the case of downregulation of E-cadherin (by expressing UAS-shg-RNAi in the AS tissue), we observed that AS cells displayed higher myosin levels at junctions, in a similar manner to ectopically stretched cells (Figure 4C and Video S8). Using our SAC analysis, we confirmed that myosin was, on average, more enriched at the cell junctions compared to WT (Figures 5A and 5B). Conversely, myosin in the medial area was less dense than in WT cells; the pulsatile foci were less intense, and pulses were arrested (Figure S5B and Video S8).

On the other hand, when we overexpressed E-cadherin (with UAS shg), we observed that cell junctions displayed an unusually low straightness (Figure 4C and Video S9). Using SAC, we observed that in this case myosin was less enriched at junctions and more present in the medial array compared to WT (Figures 4C, 5A, and 5B; Video S9). Overall, we therefore find that E-cadherin levels can directly influence junction straightness and the junctional-medial actomyosin distribution.

A Coupling of a Contraction Rate to a Straightness-Dependent Junction Removal Rate Allows for the Control of Junctional Length and Straightness

How does junctional straightness remain constant instead of decaying during tissue contraction? To get insight into the mechanisms underlying the control of straightness, we propose here a simple mathematical description of junction contraction (Figure 6A; see STAR Methods). During contraction, the time derivative of the junction straightness $s = \frac{d_v}{L}$ for a junction of length L between two vertices separated by a distance d_v, depends on the rate of contraction k_c of the vertex-vertex distance and the rate of junction removal k_j:

$$\frac{ds}{dt} = (k_j - k_c)s.$$

This indicates that a solution with constant straightness exists when $k_j = k_c$. If the two rates are constant, this description does

CellPress

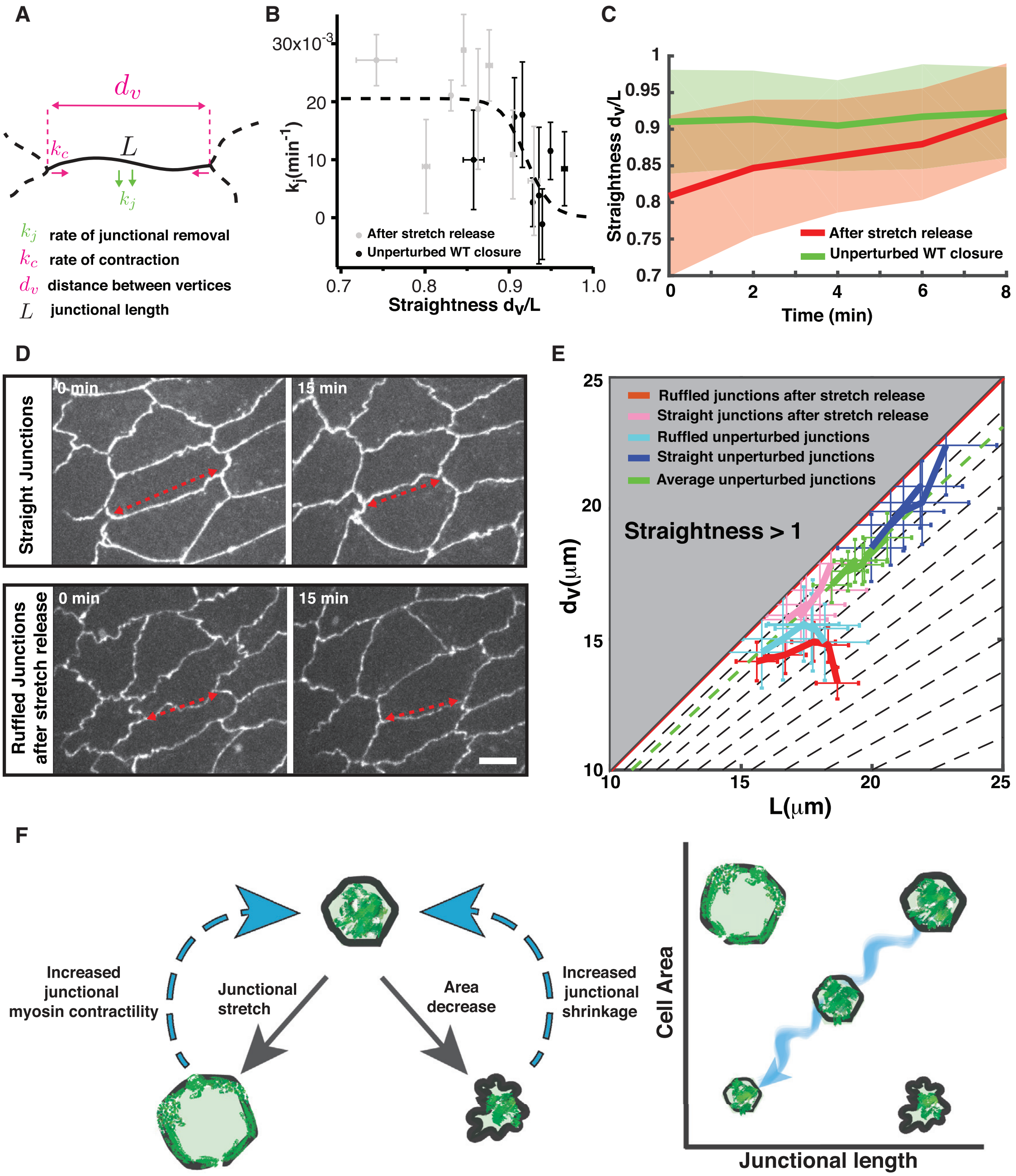

Figure 6. A Straightness Dependency of Junction Removal Rate Is Sufficient to Ensure a Constant Straightness during DC

(A) Schematic of a shrinking junction of length L with removal rate k_j, comprised between vertices at a distance d_v, and contracting with rate k_c.

(B) Graph showing the junctional removal rate k_j as a function of the straightness for AS cell adherens junctions during WT DC (black) and after stretch release (gray). The dashed line represents the sigmoid fit of the data used for our theoretical description (see STAR Methods). n = 50 junctions on 3 embryos; error bars show standard error.

(C) Junctional straightness as a function of time during WT unperturbed closure (green) and after stretch release (red). In the unperturbed case, the average junctional straightness is constant. After stretch release, the initial average straightness is lower than in the unperturbed case and converges in a few minutes toward the unperturbed straightness. Error bars show standard deviations. $N_{unperturbed}$ = 56 junctions on 4 embryos and $N_{afterstretchrelease}$ = 58 junctions on 4 embryos.

(D) Confocal images showing AS cells during DC at 15-min intervals for straight junctions (top) and ruffled junctions after stretch release (bottom). For straight junctions, we observe a coordinated reduction of the inter-vertex distance and of the junctional length, keeping an approximately constant straightness, over 15 min (red arrows). After stretch release, we observe initially ruffled junctions with an enhanced reduction in the excess length to restore a higher straightness (red arrows). Scale bar: 10 μm.

(E) Phase space of L and d_v showing iso-straightness lines as dashed lines. The gray area represents a non-physical region of the phase space corresponding to a straightness larger than 1. The red, pink, and light and dark blue lines represent experimental trajectories of ruffled and straight junctions after stretch release and

(legend continued on next page)

not result in a preferential straightness. However, if the rates k_c or k_j vary with the straightness s, this condition may determine a preferred straightness (see STAR Methods). To test whether these rates are sensitive to junction straightness in the case of AS cells, we estimated the rates of junction removal k_j and contraction k_c as functions of straightness by measuring junctional lengths L and inter-vertex distances d_v on 10–15 min intervals for junctions of different straightness (Figures 6B and S6B). We combined measurements performed on unperturbed contractile cells with measurements on cells released from external stretch (Figure 6B and S6B). In the latter case, cells appeared to have extra junctional material and reach lower straightness (Figures 6C and 6D). We observed that the dependency on straightness of the rate k_c is not clear, and the rate might be independent of straightness during WT closure (Figure S6B). The rate of junction removal, however, appears to decrease when the straightness s increases (Figures 6B and S6A). For a constant rate of contraction k_c, this indicates that when junctions are ruffled, the removal rate of junctional material increases to higher levels than the contraction rate, promoting junction straightness (Figure 6C). On the other hand, when junctions are straight, the junctional removal rate slows down to lower values than the contraction rate, promoting a reduction in straightness (Figure S6A). As a result, junction dynamics converge toward a diagonal of constant straightness in the phase space of L and d_v (Figure 6E). An additional prediction of this model is that upon perturbation, the junctional straightness will be restored on a characteristic timescale that depends on the function $k_j(s)$ (see STAR Methods). To test this, we quantified the average straightness of junctions following stress release in stretch experiments. Consistent with this simple prediction, we found that the straightness returns to WT levels on a similar timescale ($\tau_{returns}\sim$5 min) than the estimated characteristic timescale ($\tau\sim$3 min).

Overall, these results indicate that the observed control of junctional length originates in part from a dependency of the rate of junctional removal to straightness, allowing for a preferred straightness during the contraction (Figure 6F).

DISCUSSION

In this study, we have shown that adherens junction length is actively controlled during epithelial contraction, leading to a constant junctional straightness upon cell contraction. We propose that two different cellular mechanisms are at the origin of this length control. First, a modulation of actomyosin localization from the medial areas toward junctions allows for integrity maintenance by increasing actomyosin contraction along the junctions upon junctional stretch. Possibly, E-cadherin dilution triggered by junctional stretching is responsible for the myosin recruitment, a possibility supported by the anti-correlation between junctional myosin and E-cadherin (Figure 4B). Second, the junction removal rate depends on junctional straightness, maintaining a preferred junctional straightness. Presumably, the endocytosis machinery is at the origin of this maintenance (Figure 6F). As junctional tension is dependent on junctional straightness (Figure 1E), maintaining junctional straightness could also allow it to maintain junctional tension, which remains approximately constant during DC (Saias et al., 2015).

These mechanisms rely on a complex interplay between junctional E-cadherin, actomyosin, and tension. Indeed, we find that (1) higher junctional E-cadherin levels are associated with lower myosin levels (Figure 4B) and (2) decreased junctional straightness (Figure 4C) is associated with lower junctional tension (Figure 1E). These observations suggest that E-cadherin is an essential component for the control of adherens junction length and for actomyosin localization in the cell. In cell culture experiments, E-cadherin has already been determined to be a key player in cell-cell mechanical interplay and shown to interact closely with the actomyosin cortex (Borghi and James Nelson, 2009; Borghi et al., 2012; Ratheesh et al., 2012; Yap et al., 2018). In the case of the AS tissue, we observe an enrichment of actomyosin at junctions when E-cadherin levels are reduced. A similar interaction has been previously identified in the context of cytokinesis (Pinheiro et al., 2017). Our results indicate a general interplay between E-cadherin and myosin that could play a role in several morphogenetic rearrangements. Such an interplay is likely to involve actin-associated proteins that could respond to changes in mechanical states of the adherens junction. Consistent with this idea, previous studies identified dynamic enrichment of the formin Diaphanous or of vinculin specifically at adherens junctions (Hara et al., 2016; Homem and Peifer, 2008).

Interestingly, we observed that when junctions are stretched and E-cadherin density decreased, the contractile pulses of the AS tissue are arrested, possibly due to the observed myosin recruitment to junctions. Such pulses of contraction have been identified in several tissues during *Drosophila*, chicken, and mouse embryo development (Maître et al., 2015; Martin et al., 2009; Rauzi et al., 2010; Skoglund et al., 2008). They have been associated with global tissue contraction and epithelial remodeling. Here, we have shown that the pulsatile activity can be tuned by junctional stretch and E-cadherin levels. Our results therefore uncover a direct coupling between cell-cell adhesion and the contractile activity of the cell.

Finally, we found that the rate of junction removal appears to depend on junctional straightness. How is junction straightness sensed to dictate junction removal? Laser ablation experiments

in unperturbed closure, respectively. The green line represents the average junction trajectory in unperturbed closure, following a constant straightness (green dashed line). Ruffled junctions are junctions with an initial straightness lower than the mean straightness minus a standard deviation on a total sample of 56 and 58 junctions for unperturbed and after stretch release, respectively. Straight junctions are junctions with an initial straightness larger than the mean straightness plus a standard deviation. Error bars show standard errors. $N_{ruffled\_unperturbed}$ = 15 junctions, $N_{ruffled\_afterstretchrelease}$ = 28 junctions, $N_{straight\_unperturbed}$ = 14 junctions, $N_{straight\_afterstretchrelease}$ = 11 junctions, and $N_{average\_unperturbed}$ = 56 junctions on 4 embryos.

(F) Schematic showing mechanisms underlying the control of junctional length and straightness during dorsal closure (Left) and its consequence on the concerted reduction in cell size and junctional length (Right). The schematic on the left shows that (1) when excess junctional length and ruffles are present, enhanced junctional removal takes place and restores junctional straightness, and (2) when junctions are stretched, actomyosin relocalizes from the cell medial array to the junctions to reduce junctional length. The schematic on the right shows that these two mechanisms allow a coordinated reduction of cell area and junctional length during tissue contraction. In the phase space of cell area and junctional length, the two mechanisms allow the cells to progress along the diagonal during epithelial contraction by preserving them to deviate toward the upper left side where junctions are stretched or toward the lower right side where junctions are ruffled.

indicate that junctions with lower straightness also have lower mechanical tension (Figure 1E). In single cells, it is known that the endocytic rate is modulated by the tension of the cell membrane (Apodaca, 2002; Raucher and Sheetz, 1999; Sheetz, 2001). In a developmental context, endocytosis could also be tension sensitive, resulting in the control of junctional straightness through junctional tension. Alternatively, E-cadherin density could play a role in the control of the junction turnover rate. Indeed, an increase of E-cadherin density associated to a decrease in junction straightness could trigger an enhanced endocytic rate, therefore controlling junction straightness and tension.

The mechanisms regulating junction length and integrity identified here involve the activity of two essential cellular machineries. They are therefore likely to act during many morphogenetic events that involve tissue contraction, such as neural tube closure (Nishimura et al., 2012), optic cup morphogenesis (Nicolás-Pérez et al., 2016; Sidhaye and Norden, 2017), or wound healing (Hinz and Gabbiani, 2003).

STAR★METHODS

Detailed methods are provided in the online version of this paper and include the following:

- KEY RESOURCES TABLE
- CONTACT FOR REAGENT AND RESOURCE SHARING
- EXPERIMENTAL MODEL AND SUBJECT DETAILS
  - *Drosophila* Strains
  - Sample Preparation and Imaging
- METHOD DETAILS
  - Laser Nanosurgery
  - Methodology for Stretching Experiments
  - Cell and Junctions Segmentation
  - Scaled Average Cell Analysis
- QUANTIFICATION AND STATISTICAL ANALYSIS
  - Theory

SUPPLEMENTAL INFORMATION

Supplemental Information includes six figures and nine videos and can be found with this article online at https://doi.org/10.1016/j.devcel.2018.10.025.

ACKNOWLEDGMENTS

We thank Anna Oddone and Cristina Pujadas for discussions and critical reading of the manuscript. We thank Enrique Martin Blanco, Yang Hong, and Damian Brunner for fly lines. We are grateful to the ALMU team for providing help with the microscopy. The research leading to the results has received funding from the Spanish Ministry of Economy, Industry and Competitiveness (MEIC) to the EMBL partnership and Centro de Excelencia Severo Ochoa and to the Plan Nacional, BFU2010-16546 and BFU2015-68754. We acknowledge the support of the CERCA Programme/Generalitat de Catalunya. Guillaume Salbreux was supported by the Francis Crick Institute which receives its core funding from Cancer Research UK (FC001317), the UK Medical Research Concil (FC001317) and the Wellcome Trust (FC001317).

AUTHOR CONTRIBUTIONS

Conceptualization, A.S., P.H., K.D., A.D., and J.S.; Methodology, A.S., P.H., K.D., A.D., G.S., and J.S.; Formal Analysis and Software, P.H., K.D., G.S., and J.S.; Investigation, A.S. and A.D.; Resources, J.C. and J.S.; Writing – Original Draft, J.S.; Writing – Review & Editing, A.S., P.H., A.D., K.D., G.S., and J.S.; Supervision, K.D. and J.S.; Funding Acquisition, J.S.

DECLARATION OF INTERESTS

The authors declare no competing interests.

Received: February 12, 2018
Revised: September 25, 2018
Accepted: October 22, 2018
Published: November 19, 2018

REFERENCES

Aigouy, B., Farhadifar, R., Staple, D.B., Sagner, A., Röper, J.C., Jülicher, F., and Eaton, S. (2010). Cell flow reorients the axis of planar polarity in the wing epithelium of Drosophila. Cell *142*, 773–786.

Apodaca, G. (2002). Modulation of membrane traffic by mechanical stimuli. Am. J. Physiol. Renal Physiol. *282*, F179–F190.

Borghi, N., and James Nelson, W. (2009). Intercellular adhesion in morphogenesis: molecular and biophysical considerations. Curr. Top. Dev. Biol. *89*, 1–32.

Borghi, N., Sorokina, M., Shcherbakova, O.G., Weis, W.I., Pruitt, B.L., Nelson, W.J., and Dunn, A.R. (2012). E-cadherin is under constitutive actomyosin-generated tension that is increased at cell-cell contacts upon externally applied stretch. Proc. Natl. Acad. Sci. USA *109*, 12568–12573.

Bucci, C., Parton, R.G., Mather, I.H., Stunnenberg, H., Simons, K., Hoflack, B., and Zerial, M. (1992). The small GTPase rab5 functions as a regulatory factor in the early endocytic pathway. Cell *70*, 715–728.

Colombelli, J., and Solon, J. (2013). Force communication in multicellular tissues addressed by laser nanosurgery. Cell Tissue Res. *352*, 133–147.

Farhadifar, R., Röper, J.C., Aigouy, B., Eaton, S., and Jülicher, F. (2007). The influence of cell mechanics, cell-cell interactions, and proliferation on epithelial packing. Curr. Biol. *17*, 2095–2104.

Gorfinkiel, N., Blanchard, G.B., Adams, R.J., and Martinez Arias, A. (2009). Mechanical control of global cell behaviour during dorsal closure in Drosophila. Development *136*, 1889–1898.

Hara, Y., Shagirov, M., and Toyama, Y. (2016). Cell boundary elongation by non-autonomous contractility in cell oscillation. Curr. Biol. *26*, 2388–2396.

Hayes, P., and Solon, J. (2017). Drosophila dorsal closure: an orchestra of forces to zip shut the embryo. Mech. Dev. *144*, 2–10.

Hinz, B., and Gabbiani, G. (2003). Cell-matrix and cell-cell contacts of myofibroblasts: role in connective tissue remodeling. Thromb. Haemost. *90*, 993–1002.

Homem, C.C., and Peifer, M. (2008). Diaphanous regulates myosin and adherens junctions to control cell contractility and protrusive behavior during morphogenesis. Development *135*, 1005–1018.

Huang, J., Zhou, W., Dong, W., Watson, A.M., and Hong, Y. (2009). From the Cover: directed, efficient, and versatile modifications of the Drosophila genome by genomic engineering. Proc. Natl. Acad. Sci. USA *106*, 8284–8289.

Hutson, M.S., Tokutake, Y., Chang, M.S., Bloor, J.W., Venakides, S., Kiehart, D.P., and Edwards, G.S. (2003). Forces for morphogenesis investigated with laser microsurgery and quantitative modeling. Science *300*, 145–149.

Jacinto, A., Woolner, S., and Martin, P. (2002). Dynamic analysis of dorsal closure in Drosophila: from genetics to cell biology. Dev. Cell *3*, 9–19.

Kiehart, D.P., Galbraith, C.G., Edwards, K.A., Rickoll, W.L., and Montague, R.A. (2000). Multiple forces contribute to cell sheet morphogenesis for dorsal closure in Drosophila. J. Cell Biol. *149*, 471–490.

Lecuit, T., Lenne, P.F., and Munro, E. (2011). Force generation, transmission, and integration during cell and tissue morphogenesis. Annu. Rev. Cell Dev. Biol. *27*, 157–184.

Levayer, R., Pelissier-Monier, A., and Lecuit, T. (2011). Spatial regulation of Dia and myosin-II by RhoGEF2 controls initiation of E-cadherin endocytosis during epithelial morphogenesis. Nat. Cell Biol. *13*, 529–540.

Llimargas, M., and Casanova, J. (2010). Apical constriction and invagination: a very self-reliant couple. Dev. Biol. *344*, 4–6.

Maître, J.L., Niwayama, R., Turlier, H., Nédélec, F., and Hiiragi, T. (2015). Pulsatile cell-autonomous contractility drives compaction in the mouse embryo. Nat. Cell Biol. *17*, 849–855.

Martin, A.C., and Goldstein, B. (2014). Apical constriction: themes and variations on a cellular mechanism driving morphogenesis. Development *141*, 1987–1998.

Martin, A.C., Kaschube, M., and Wieschaus, E.F. (2009). Pulsed contractions of an actin-myosin network drive apical constriction. Nature *457*, 495–499.

Mateus, A.M., Gorfinkiel, N., Schamberg, S., and Martinez Arias, A. (2011). Endocytic and recycling endosomes modulate cell shape changes and tissue behaviour during morphogenesis in Drosophila. PLoS One *6*, e18729.

Nicolás-Pérez, M., Kuchling, F., Letelier, J., Polvillo, R., Wittbrodt, J., and Martínez-Morales, J.R. (2016). Analysis of cellular behavior and cytoskeletal dynamics reveal a constriction mechanism driving optic cup morphogenesis. Elife *5*, e15797.

Niessen, C.M., Leckband, D., and Yap, A.S. (2011). Tissue organization by cadherin adhesion molecules: dynamic molecular and cellular mechanisms of morphogenetic regulation. Physiol. Rev. *91*, 691–731.

Nishimura, T., Honda, H., and Takeichi, M. (2012). Planar cell polarity links axes of spatial dynamics in neural-tube closure. Cell *149*, 1084–1097.

Pinheiro, D., Hannezo, E., Herszterg, S., Bosveld, F., Gaugue, I., Balakireva, M., Wang, Z., Cristo, I., Rigaud, S.U., Markova, O., et al. (2017). Transmission of cytokinesis forces via E-cadherin dilution and actomyosin flows. Nature *545*, 103–107.

Ratheesh, A., Gomez, G.A., Priya, R., Verma, S., Kovacs, E.M., Jiang, K., Brown, N.H., Akhmanova, A., Stehbens, S.J., and Yap, A.S. (2012). Centralspindlin and alpha-catenin regulate Rho signalling at the epithelial zonula adherens. Nat. Cell Biol. *14*, 818–828.

Raucher, D., and Sheetz, M.P. (1999). Membrane expansion increases endocytosis rate during mitosis. J. Cell Biol. *144*, 497–506.

Rauzi, M., Lenne, P.F., and Lecuit, T. (2010). Planar polarized actomyosin contractile flows control epithelial junction remodelling. Nature *468*, 1110–1114.

Rauzi, M., Verant, P., Lecuit, T., and Lenne, P.F. (2008). Nature and anisotropy of cortical forces orienting Drosophila tissue morphogenesis. Nat. Cell Biol. *10*, 1401–1410.

Saias, L., Swoger, J., D'Angelo, A., Hayes, P., Colombelli, J., Sharpe, J., Salbreux, G., and Solon, J. (2015). Decrease in cell volume generates contractile forces driving dorsal closure. Dev. Cell *33*, 611–621.

Sawyer, J.M., Harrell, J.R., Shemer, G., Sullivan-Brown, J., Roh-Johnson, M., and Goldstein, B. (2010). Apical constriction: a cell shape change that can drive morphogenesis. Dev. Biol. *341*, 5–19.

Sheetz, M.P. (2001). Cell control by membrane-cytoskeleton adhesion. Nat. Rev. Mol. Cell Biol. *2*, 392–396.

Sidhaye, J., and Norden, C. (2017). Concerted action of neuroepithelial basal shrinkage and active epithelial migration ensures efficient optic cup morphogenesis. Elife *6*, e22689.

Skoglund, P., Rolo, A., Chen, X., Gumbiner, B.M., and Keller, R. (2008). Convergence and extension at gastrulation require a myosin IIB-dependent cortical actin network. Development *135*, 2435–2444.

Solon, J., Kaya-Copur, A., Colombelli, J., and Brunner, D. (2009). Pulsed forces timed by a ratchet-like mechanism drive directed tissue movement during dorsal closure. Cell *137*, 1331–1342.

Yap, A.S., Duszyc, K., and Viasnoff, V. (2018). Mechanosensing and mechanotransduction at cell-cell junctions. Cold Spring Harb. Perspect. Biol. *10*, a028761.

STAR★METHODS

KEY RESOURCES TABLE

REAGENT or RESOURCE	SOURCE	IDENTIFIER
Experimental Models: Organisms/Strains		
DE Cad-Tomato, Sqh-GFP, c332.3 Gal4	This study	N/A
Sqh-GFP::C381 Gal4	Damian Brunner Lab	N/A
E Cad-GFP::C381 Gal4	Damian Brunner Lab	N/A
UAS mCherry Moesin	Damian Brunner Lab	N/A
P{UAS-Rab5.S43N}2	Enrique Martin Blanco Lab	RRID:Flybase_ FBti0150344
UAS Shg-R	Bloomington stock center	RRID:BDSC_58494
UAS Shg RNAi	Bloomington stock center	RRID:BDSC_32904
c381 Gal4	Bloomington stock center	RRID:BDSC_3734
Software and Algorithms		
Fiji	Fiji	https://fiji.sc
Python	Python	https://www.python.org/

CONTACT FOR REAGENT AND RESOURCE SHARING

Further information and requests for resources and reagents should be directed to and will be fulfilled by the Lead Contact, Jérôme Solon (jerome.solon@crg.es).

EXPERIMENTAL MODEL AND SUBJECT DETAILS

Drosophila Strains

The following lines were used: *w*; DE Cad-Tomato, Sqh-GFP*, *C332.3 Gal4* (generated in this study, Cad-Tomato was a gift from Hong Y Lab [Huang et al., 2009]), *w*; Sqh-GFP;;c381-Gal4*, *w*;E Cad-GFP;;c381-Gal4, w*;UAS Shg-R (Bloomington 58494), UAS Shg RNAi(Bloomington 32904) , w*;P{UAS-Rab5.S43N}2* (gift from Enrique Martin Blanco lab), *UAS-mCherry-Moesin (gift from Damian Brunner Lab) and w, c381-Gal4 (Bloomington 3734)*.

Sample Preparation and Imaging

Embryos were collected at 25 degrees overnight. They were then dechorionated with 50% bleach and washed with water. Embryos at the right stage were chosen under the fluorescent lamp and mounted depending on the experiment to be performed. For confocal imaging, the embryos were mounted on a glass-bottomed culture dish (Mattek), covered with 1% agarose and immersed in 1% PBS. For stretching experiments, the embryos were mounted on a glass-bottomed culture dish (Mattek) that had been coated with heptane glue, and immersed in 1% PBS. The imaging was carried using a Yokogawa CSU-X1 spinning disk on an inverted Olympus 1X81 microscope, with lens U plan S Apo 60x 1.45 Oil.

METHOD DETAILS

Laser Nanosurgery

Laser ablation was performed with a scanned 355-nm pulsed (470 ps)-laser, coupled through the port of an AxioVert 200 M and focused through a Zeiss C-Apo 63x/1.2 W lens. Fluorescence confocal images were acquired through a custom spinning disk unit coupled to the inverted microscope, with the excitation line at 488 nm of an Argon laser. Simultaneous fluorescence and transmission imaging was performed with two identical charge-coupled device cameras and a 50/50 splitter internal to the microscope stand. Fluorescence imaging was done at the rate of around 1-3 images/sec. For the dissection of the cell-cell junction, initial recoil velocities were measured by tracking the tip of the dissected junctions and calculating the 2D speed of the traces (Figures 1D and S1A). This allows us to estimate initial retraction velocity for the cut ends of straight and convoluted junctions. Similar analysis was performed on the displacement of the vertices in contact with the dissected junction (Figure S1A). To extract initial retraction velocity, the profile of spatial relaxation was automatically calculated from the path length of the tracks and fitted with a single exponential using a Python script. The initial retraction velocity V_0 was calculated from the fitting function f(t) as follows:

$$f(t) = A \cdot \left(1 - e^{-\frac{t}{\tau}}\right),$$

$$V_0 = \frac{A}{\tau},$$

where A is the total retraction amplitude and τ the decay rate constant of the exponential.

Methodology for Stretching Experiments

For force application experiments, a round cover glass (5mm in diameter) was fixed to the end of a glass capillary (1 × 90mm) with super glue. The capillary was held with a micromanipulator (Narashige – Model UMM-3FC) and compression on embryos was applied by manually displacing the position of the coverslip in Z using the coarse and fine control knobs. Force amplitude was not controlled; instead the position of the coverslip was adjusted to obtain about 10-20% cell surface increase.

Cell and Junctions Segmentation

Amnioserosa cells were segmented and tracked in a semi-automated manner. Cells that remained in the field of view during the entire stretching experiment were selected and segmented using the software Packing Analyzer V2.0 (Aigouy et al., 2010). Manual corrections were performed on each frame to ensure proper segmentation of the selected cells (Figure S1D). Upon segmentation, cells were individually tracked with custom-made software written in Python. In this way, data on each cell including the localization of myosin and E-cadherin intensities, cell area, perimeter, and other measures were extracted over time. Area was calculated as the sum of pixels included in the segmented area multiplied by the area covered by a pixel. Data on junction path length (L) and inter-vertex distances (d_v) was extracted manually using FIJI.

Scaled Average Cell Analysis

The average cell analysis was performed using custom software written in Python. Segmented images were aligned along the AP axis. From the center of mass of each cell, we performed a radial analysis to detect the position of the cell boundaries and myosin localization. For each angle, an average position of the boundary was calculated to generate the average contour of a cell. Then, after a background removal (tophat filtering with a rolling ball of radius 50 pixels), the distribution of myosin from the cell centroid to the junction at each angle, was scaled along the appropriate centroid-junction length on the average cell. This operation was performed on each single cell, before averaging across cells (Figure S1E). In Figures 2E and 3E, average cells were generated at each time point during and after stretch on a sample of 58 to 70 cells. In Figure 4A, average cells were generated on individual cells over a 5-min time frame with a 10s frame rate. The correlation between myosin and E-cadherin levels in Figure 4B was obtained by analyzing only pixels close to the boundary ($\leqq$1.5 pixels from the edge) of the individual cells averaged across time points (see for example Figure 4A).

QUANTIFICATION AND STATISTICAL ANALYSIS

The statistical details of experiments are described in the figure legends, including the number of embryo, the number of cells used for each analysis and the standard deviations or standard error to the mean.

Theory

Here, we discuss a simple description of coupled dynamics of junction removal and tissue contraction. We consider a single cell-cell junction as a path of length L between two vertices separated by the distance d_v. The dynamics of the vertex-vertex distance and of the path length are described by the following system of equations:

$$\frac{dd_v}{dt} = -k_c d_v$$

$$\frac{dL}{dt} = -k_j L.$$

Here, k_c is a contraction rate which we expect to be equal to the rate of tissue contraction on average, and k_j is a rate of junction removal.

Combining these two equations, the straightness of the junction $S = \frac{d_v}{L}$ changes according to $\frac{ds}{dt} = (k_j - k_c)s$.

If k_j and k_c are constant and $k_j \neq k_c$, the equation above yields exponential growth or decay of the straightness, $s(t) = s(0)e^{(k_j - k_c)t}$, such that $s \rightarrow 0$ for $k_j < k_c$ (low rate of junctional removal), and $s \rightarrow \infty$ for $k_j > k_c$ (high rate of junction removal). In the second case, geometrical constraints impose that $s \leq 1$, so that the assumption that the rate k_j and k_c are constant must break down in this limit.

Experimentally, during dorsal closure, we obsere that the straightness of junctions is approximately constant (see Figure 1C) and smaller than 1. According to the previous equation, a constant straightness imposes $k_j = k_c$. For arbitrary constant values of k_j and k_c, this condition will not be met in general. Amechanism must therefore ensure that the rates k_j or k_c can adjust to meet this condition.

We find indeed that the rate of junction removal k_j appears to vary with the junction straightness (Figure 6B). One can fit the straightness dependency of k_j with a decreasing sigmoid function:

$$k_j(s) = \frac{k_j^0}{e^{r(s-s_0)}+1},$$

with $k_j^0 = 0.0205 min^{-1}$, $s_0 = 0.9202$ and $r = 68.3$.

We also quantified the rate of contraction k_c as a function of straightness. The dependency of k_c to the straightness appears less clear (see Figure S6B) and looks roughly independent on straightness. We therefore consider here the average rate of contraction $k_c = 0.0091 \pm 0.044 min^{-1}$.

The steady-state straightness $s = s^*$ is then set by the condition

$$k_j(s^*) = k_c,$$

which is the point at which straightness decrease due to tissue contraction is balanced by the straightness increase by junction removal.

Solving for this last equation, we obtain a steady-state straightness $s^* = 0.923 \pm 0.127$. This value is in line with the average junction straightness measured in the same conditions (Figure 6C).

Finally, we note that linearising the differential equation for the straightness around the fixed point s^*, we obtain

$$\frac{d(s-s^*)}{dt} = k_j'(s^*)s^*(s-s^*),$$

so that the straightness responds to perturbations on a characteristic time scale $\tau = -\frac{1}{k_j'(s^*)s^*}$. Using the sigmoid fitting function introduced above for $k_j(s)$, we find that for the WT, $\tau \simeq 3.1\ min$. This time is indeed roughly the timescale necessary to restore junction straightness following a stretch release experiment (Figure 6C).

Developmental Cell, Volume *47*

Supplemental Information

Adherens Junction Length during Tissue Contraction

Is Controlled by the Mechanosensitive Activity

of Actomyosin and Junctional Recycling

Angughali Sumi, Peran Hayes, Arturo D'Angelo, Julien Colombelli, Guillaume Salbreux, Kai Dierkes, and Jérôme Solon

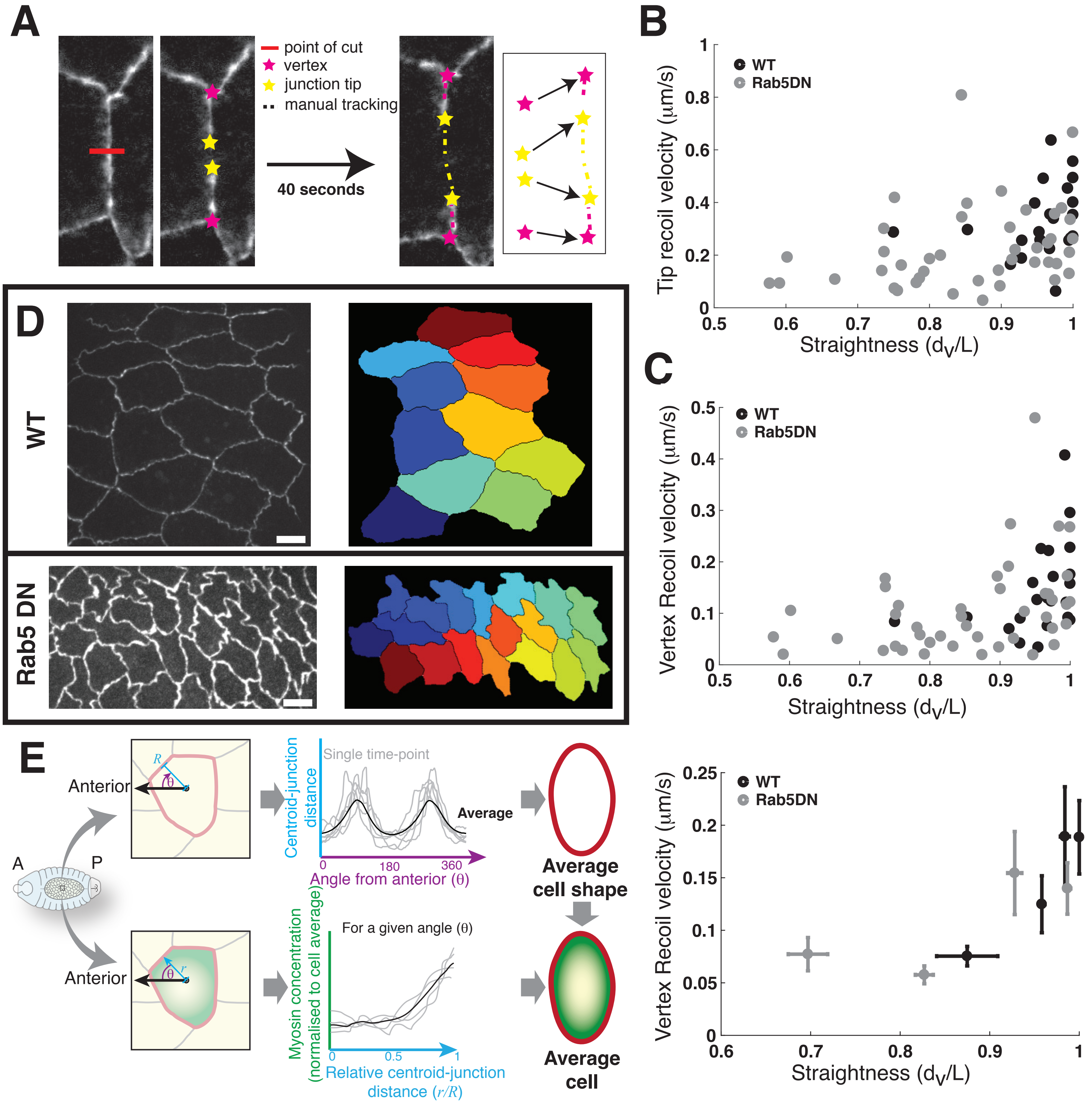
A
point of cut
vertex
junction tip
manual tracking
40 seconds
B
WT
Rab5DN
Tip recoil velocity (μm/s)
Straightness (dV/L)
C
WT
Rab5DN
Vertex Recoil velocity (μm/s)
Straightness (dV/L)
D
WT
Rab5 DN
E
Anterior
R
θ
A
P
Centroid-junction distance
Single time-point
Average
0
180
360
Angle from anterior (θ)
Average cell shape
r
Myosin concentration (normalised to cell average)
For a given angle (θ)
0
0.5
1
Relative centroid-junction distance (r/R)
Average cell
WT
Rab5DN
Vertex Recoil velocity (μm/s)
Straightness (dV/L)

Figure S1. Methodologies for laser dissection, cell segmentation and Average cell generation (related to figure 1 and Figure 2).

(A) Schematic explaining the methodology used to extract the initial recoil velocity from laser dissection experiments. Tips of junctions and vertices are tracked manually after laser dissection over a time period of 40 seconds, to extract the distance from their starting position as function of time. Onto this, an exponential fit is performed to extract the initial recoil velocity.

(B) Graph showing the initial recoil velocity after laser dissection of tips of junctions as a function of the straightness of the junction before laser dissection for WT and Rab5DN expressing cells. We observe an increase in recoil velocity when reaching a straightness close to 1. Each point is for an individual junction, and shows the average of the initial recoil velocities of the two cut ends.

(C) (Top) Graph showing the initial recoil velocity after laser dissection of the vertices of dissected junctions as a function of the straightness of the junction before laser dissection for WT and Rab5DN expressing cells. We observe an increase in recoil velocity when reaching a straightness close to 1. Each point shows the average of the initial recoil velocities of the two vertices of an individual junction. (Bottom) Graph showing the averaged initial recoil velocity of vertices after laser dissection corresponding to the graph above. Each point is an average of 5-6 junctions for WT and 10-11 junctions for Rab5DN. Error bars show standard errors of the mean. N_{WT}=22 junctions and N_{Rab5DN}=43 junctions.

(D) Images showing examples of WT and Rab5DN expressing AS cells tagged with Tomato-Ecadherin and their respective segmentation performed using Packing Analyzer V2.0 and a Python script (see methods).

(E) Schematic explaining the methodology to generate an average cell. The cells are oriented along the AP axis. With a radial scanning, the distance *R* between the cell contour and the center of mass is detected for each angle , to reconstruct an average contour (Top). Using the same radial scan, myosin levels along the line *r* from the center of mass cell to the cell contour are detected and an average for each angle is generated and scaled onto the average contour.

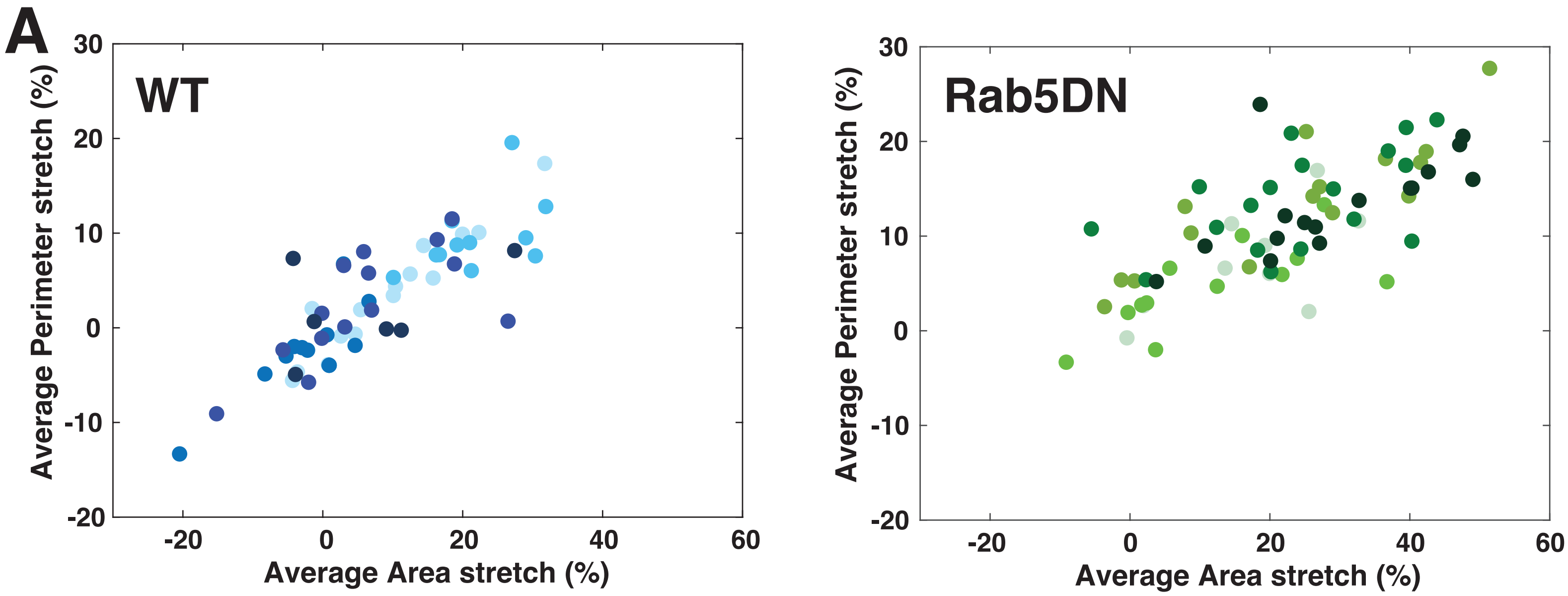

	Area stretch	Perimeter stretch
Embryo 1	9.5 ± 10 %	3.5 ± 6 %
Embryo 2	22 ± 7 %	9.5 ± 4 %
Embryo 3	-2.1 ± 7.1 %	-2.5 ± 4.7 %
Embryo 4	6 ± 11 %	2.4 ± 6 %
Embryo 5	6.5 ± 12.2 %	1.8 ± 5 %

	Area stretch	Perimeter stretch
Embryo 1	17.2 ± 11 %	7.2 ± 5.6 %
Embryo 2	12 ±13.5 %	4.6 ± 4.7 %
Embryo 3	23.3 ± 17.6 %	13.5 ± 6.8 %
Embryo 4	23.9 ± 13.7 %	13.8 ± 5.2 %
Embryo 5	29.7 ± 13.7 %	13.4 ± 5.1 %

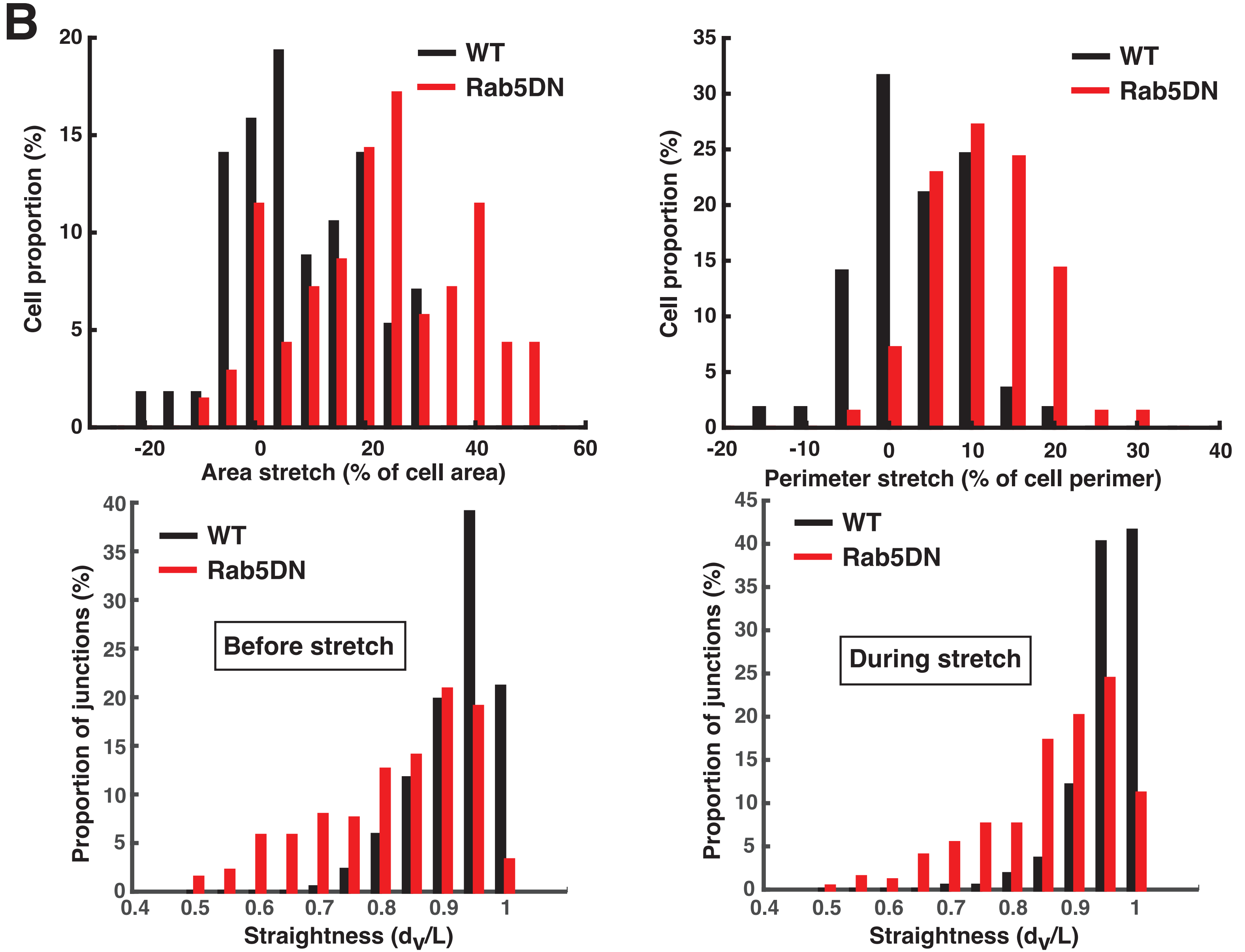

Figure S2. Amplitude of stretch performed by squeezing embryos (related to figure 2 and 3).

(A) (Top) Graphs showing the average perimeter stretch as a function of the average area stretch during our squeezing experiments for WT (Left) and Rab5DN expressing cells (Right). Average area/perimeter stretch was calculated as the average area/perimeter within the first minute after the stretch onset divided by the average area/perimeter over the last 10-15 minutes before stretch. Each dot represents one cell and each color, one embryo (corresponding to the embryos in the tables below). (Bottom) Tables showing the average stretch and standard deviation per embryo analyzed for WT (Left) and Rab5DN expressing cells (Right).

(B) (Top-Left) Distribution of the increase in average area per cell obtained during stretch experiments for WT and Rab5DN expressing cells. In WT, we generate an average area increase of $8.4 \pm 12\%$ and in Rab5, we generate an average area increase of $22 \pm 15\%$.

(Top-Right) Distribution of the increase in average perimeter per cell obtained during stretch experiments for WT and Rab5DN expressing cells. In WT, we generate an average perimeter increase of $3 \pm 6.4\%$ and in Rab5, we generate an average perimeter increase of $11 \pm 6.5\%$.

(Bottom-Left and Right) Graphs showing the distribution of junctional straightness for WT and Rab5DN expressing cells within the minute before (Left) and during (Right) stretch.

(Bottom-Left) Before stretch is applied; WT junctions exhibit an average straightness of 0.92 ± 0.06, compared to 0.82 ± 0.12 for junctions of Rab5DN expressing cells. This difference is due to the presence of increased ruffles in Rab5 DN cells.

(Bottom-Right) During stretch application; WT junctions exhibit an average straightness of 0.96 ± 0.05, and Rab5DN junctions show a straightness of 0.87 ± 0.11. In both WT and Rab5DN cells, the application of stretch increased junctional straightness. The straightness of Rab5DN junctions increased to values similar to those of WT before stretch application, however they continued to exhibit much lower straightness than the stretched WT cells. N_{WT}=224 junctions on 5 embryos and N_{Rab5DN}=279 junctions on 5 embryos.

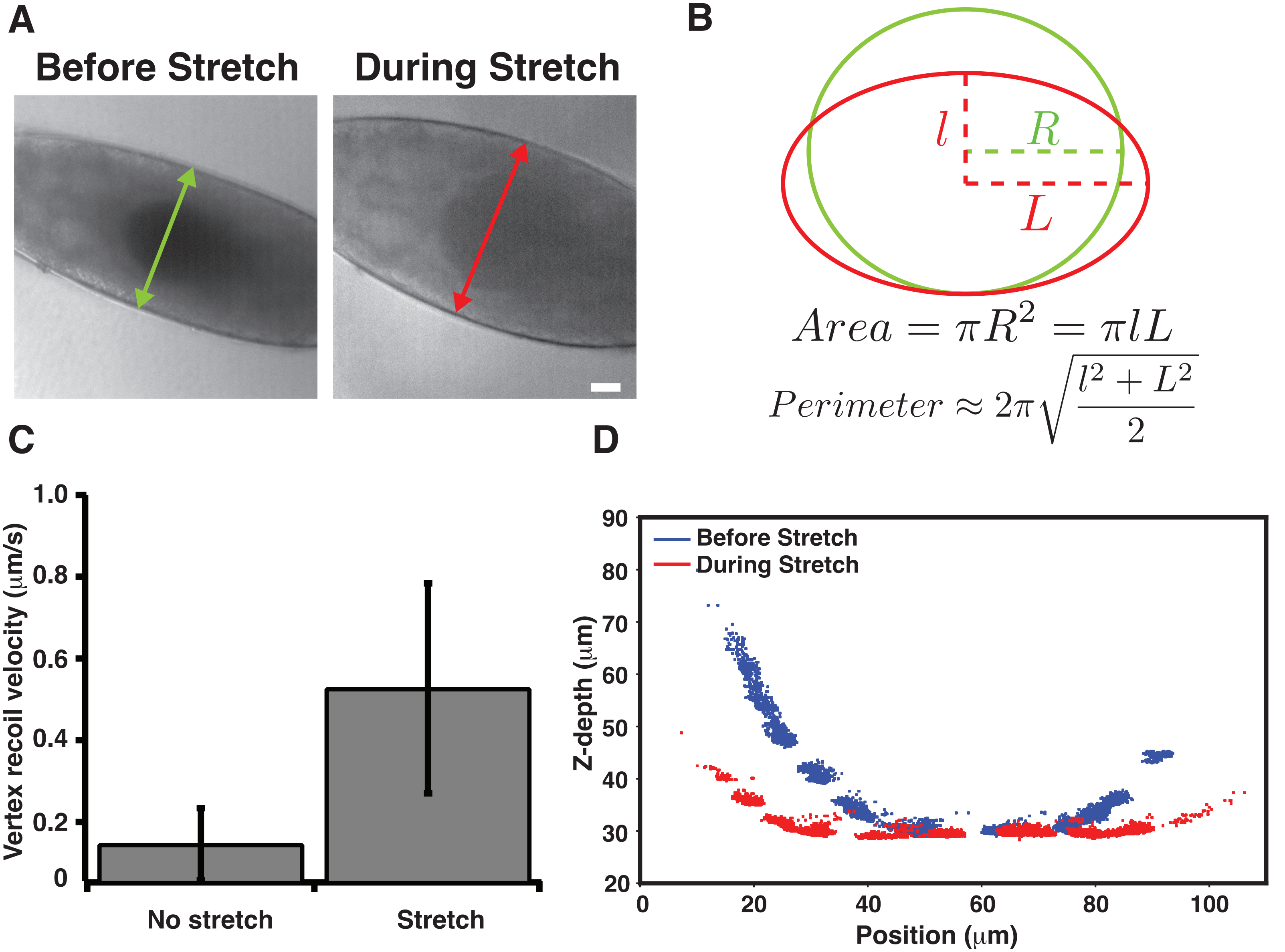
A
Before Stretch
During Stretch
B
l
R
L
$$Area = \pi R^2 = \pi l L$$
$$Perimeter \approx 2\pi \sqrt{\frac{l^2 + L^2}{2}}$$
C
Vertex recoil velocity (μm/s)
1.0
0.8
0.6
0.4
0.2
0
No stretch
Stretch
D
Before Stretch
During Stretch
Z-depth (μm)
90
80
70
60
50
40
30
20
0
20
40
60
80
100
Position (μm)

Figure S3. Embryonic deformations and stretch generated by squeezing embryos (related to figure 2)

(A) Example of an embryo imaged by transmission light before and during stretch induction. We observe an increase in width, characteristic of a change in aspect ratio. Scale bar: 40µm.

(B) Schematics showing a typical change in aspect ratio and the estimation of the change in height for a measured change in embryo width. Assuming the area of the central region of the embryo is conserved upon stretch, we calculated, from the change in embryo width, a change in height of approximately 25 % in our experiments. From this change in aspect ratio, we calculated a 8% increase in perimeter.

(C) Average vertex recoil velocity after laser dissection of WT junctions in the case where no stretch had been applied and with stretch applied. We observe an increased initial recoil velocity. $N_{NoStretch}$=22 junctions and $N_{Stretch}$=7 junctions. Error bars are standard deviations. Note that the low number in the case of stretched junctions is due to to the complexity of the experiments as embryos tend to "blast" when dissection is applied during stretch.

(D) Profiles of the central region of the embryo along the AP axis before (blue) and during (red) stretch application. We observe a change in aspect ratio from a profile nearly circular to nearly elliptic.

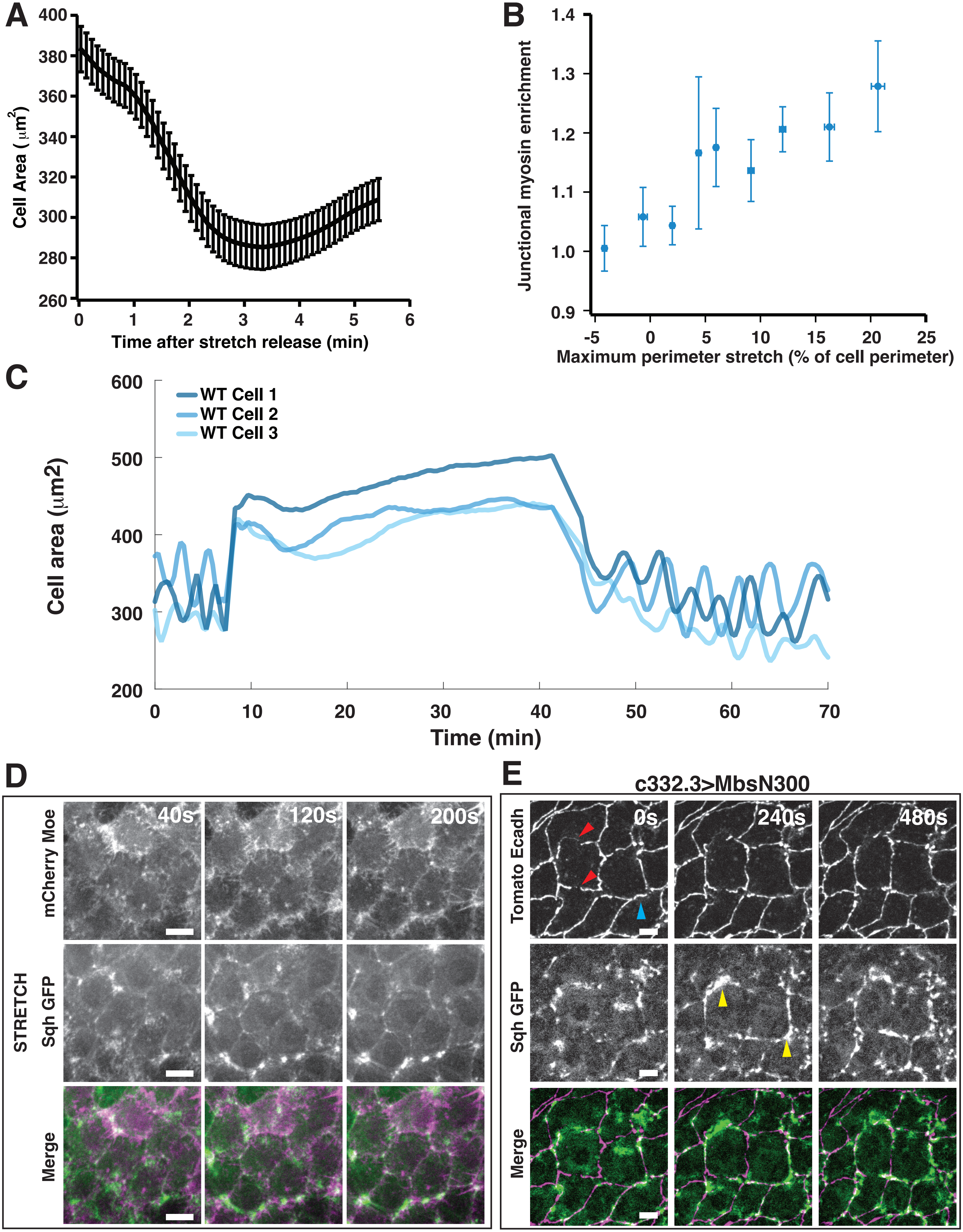

A
Cell Area (μm²)
Time after stretch release (min)
B
Junctional myosin enrichment
Maximum perimeter stretch (% of cell perimeter)
C
WT Cell 1
WT Cell 2
WT Cell 3
Cell area (μm2)
Time (min)
D
STRETCH
mCherry Moe
Sqh GFP
Merge
40s
120s
200s
E
c332.3>MbsN300
Tomato Ecadh
Sqh GFP
Merge
0s
240s
480s

Figure S4. Cellular response to ectopic stretch (related to figure 2)

(A) Average cell area over time after stress release. We observe a decrease of the area of approximately $100\mu m^2$ within the first 3 minutes. N=61 cells on 6 embryos. Error bars show standard errors.

(B) Graph showing the enrichment in myosin (defined as the ratio of the peak in myosin concentration at junctions by the initial junctional concentration) as a function of the perimeter stretch (defined as the ratio of the maximum perimeter after stretch by the average perimeter before stretch). We observe a linear correlation between the myosin enrichment and the perimeter stretch. Error bars show standard errors. N=88 cells on 7 embryos.

(C) Graph showing the apical cell surface area as a function of time for 3 individual cells before stretch, during stretch and after release. The three cells are the same as those shown in Fig2-B.

(D) Time lapse sequence showing the localization of actin (labelled with mCherry Moe) and myosin (Sqh-GFP) during stretch. We observe a progressive localization of both actin and myosin at junctions. Note that the expression of mCherry-Moe is patchy with some cells displaying lower expression due to the Gal4 driver. Scale bar: $10\mu m$.

(E) Time lapse sequence showing AS cells expressing *DE Cad-Tomato, Sqh-GFP*, *C332.3 Gal4/UAS MbsN300*. Stable gaps in adherens junctions can be observed consequent to the lower activity of myosin (Red arrowheads). Progressive disassembly of junctions can also be observed (blue arrowheads). We observe that myosin localizes to the juntional gaps but is unable to restore junctional integrity (yellow arrowheads). Scale bar: 10μm.

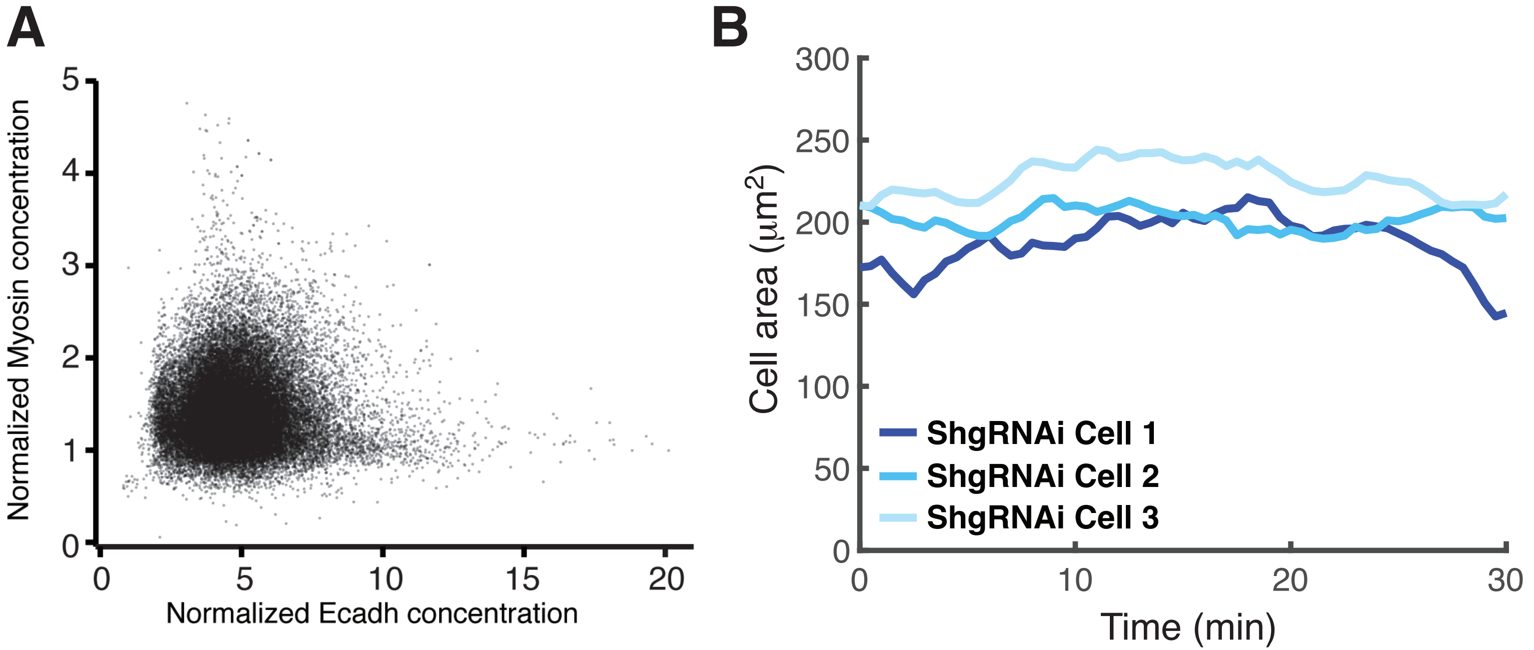
A
Normalized Myosin concentration
Normalized Ecadh concentration
0
1
2
3
4
5
0
5
10
15
20
B
Cell area (μm2)
Time (min)
300
250
200
150
100
50
0
0
10
20
30
ShgRNAi Cell 1
ShgRNAi Cell 2
ShgRNAi Cell 3

Figure S5 Adherens junctions and pulsatile activity of the AS cells during DC in WT and Shg-RNAi (related to figure 4).

(A) Graph showing the raw data that has been binned to produce the graph in Figure 4-B. Individual cells over time for 5 minutes using the SAC analysis. From each individual SAC, junctional pixels were selected, as those less than 1.5 pixels from the border, and their myosin and E-cadherin intensities (normalized by the initial value at the cell centre) were plotted against each other. Each dot therefore represents a single junctional pixel from an individual scaled average cell. We observe that pixels with the highest E-cadherin levels have the lowest myosin levels and conversely pixels with the highest myosin levels have low E-cadherin levels.

(B) Graph showing cell area over time for three individual AS cells with downregulated E-cadherin (expressing Shg-RNAi). We observe a steady area and an absence of pulsatile activity.

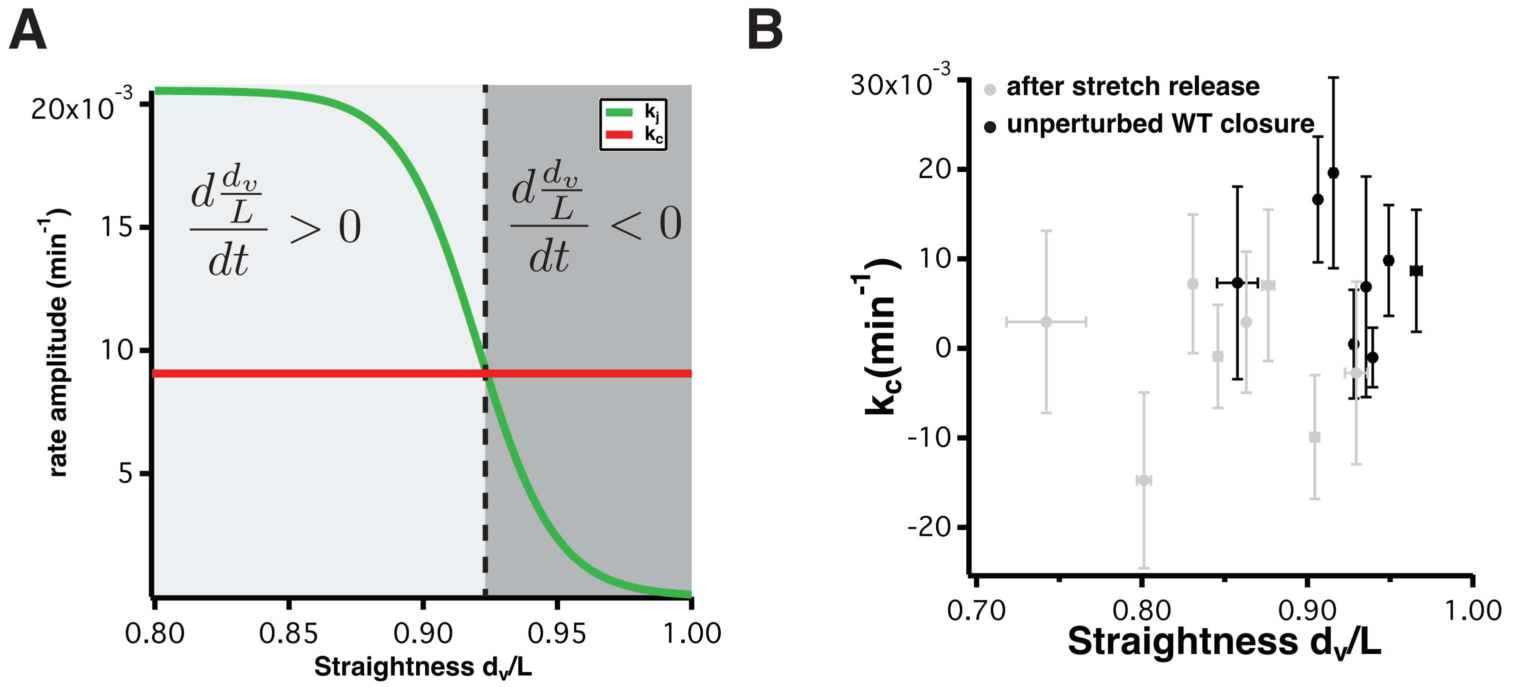

A
B
20x10^-3
15
10
5
rate amplitude (min^-1)
0.80
0.85
0.90
0.95
1.00
Straightness d_v/L
k_j
k_c
$\frac{d\frac{d_v}{L}}{dt} > 0$
$\frac{d\frac{d_v}{L}}{dt} < 0$
30x10^-3
20
10
0
-10
-20
$k_c(min^{-1})$
after stretch release
unperturbed WT closure
0.70
0.80
0.90
1.00
Straightness d_v/L

Figure S6. Straightness maintenance and rate dependencies (related to figure 6).

(A) Graph showing the fitted curve to the rate k_j as a function of the straightness, and the average value of k_c during unperturbed closure. We define two regions of low and high straightness separated by the intersection of the two curves. For low straightness, $k_j > k_c$, inducing an increase of the junction straightness, which brings the straightness back to the intersection of the two curves. For high straightness, $k_c > k_j$, inducing a reduction in straightness towards the intersection.

(B) Graph showing the contraction rate $k_c$as a function of the straightness for AS cell adherens junctions during WT DC (Black) and after stretch release (Gray). There is no clear dependency of $k_c$with straightness in the case of unperturbed closure. N=50 junctions on 3 embryos, error bars show standard errors.
